# Dual-Wavelength Photosensitive Nano-in-Micro Scaffold Regulates Innate and Adaptive Immune Responses for Osteogenesis

**DOI:** 10.1007/s40820-020-00540-z

**Published:** 2020-11-21

**Authors:** Qin Zhao, Miusi Shi, Chengcheng Yin, Zifan Zhao, Jinglun Zhang, Jinyang Wang, Kailun Shen, Lingling Zhang, Hua Tang, Yin Xiao, Yufeng Zhang

**Affiliations:** 1 The State Key Laboratory Breeding Base of Basic Science of Stomatology (Hubei-MOST) and Key Laboratory of Oral Biomedicine, Ministry of Education, School and Hospital of Stomatology, Wuhan University, Wuhan, 430079 People’s Republic of China; 2 Medical Research Institute, School of Medicine, Wuhan University, Wuhan, 430071 People’s Republic of China; 3 Institute of Immunology, Shandong First Medical University & Shandong Academy of Medical Sciences, Taian, 271000 People’s Republic of China; 4 Institute of Health and Biomedical Innovation & Australia-China Centre for Tissue Engineering and Regenerative Medicine, Queensland University of Technology, Kelvin Grove, 4059 QLD Australia

**Keywords:** Gold nanocage, Drug release, Photocatalysis, Immunomodulation, Osteoinduction

## Abstract

The immune response of a biomaterial determines its osteoinductive effect. Although the mechanisms by which some immune cells promote regeneration have been revealed, the biomaterial-induced immune response is a dynamic process involving multiple cells. Currently, it is challenging to accurately regulate the innate and adaptive immune responses to promote osteoinduction in biomaterials. Herein, we investigated the roles of macrophages and dendritic cells (DCs) during the osteoinduction of biphasic calcium phosphate (BCP) scaffolds. We found that osteoinductive BCP directed M2 macrophage polarization and inhibited DC maturation, resulting in low T cell response and efficient osteogenesis. Accordingly, a dual-targeting nano-in-micro scaffold (BCP loaded with gold nanocage, BCP-GNC) was designed to regulate the immune responses of macrophages and DCs. Through a dual-wavelength photosensitive switch, BCP-GNC releases interleukin-4 in the early stage of osteoinduction to target M2 macrophages and then releases dexamethasone in the later stage to target immature DCs, creating a desirable inflammatory environment for osteogenesis. This study demonstrates that biomaterials developed to have specific regulatory capacities for immune cells can be used to control the early inflammatory responses of implanted materials and induce osteogenesis.

**Electronic supplementary material:**

The online version of this article (https://doi.org/10.1007/s40820-020-00540-z) contains supplementary material, which is available to authorized users.

## Highlights

- The controlled release of IL-4 and dexamethasone by a dual-wavelength photosensitive nano-in-micro scaffold (biphasic calcium phosphate loaded with goad nanocage, BCP-GNC) enables the regulation of specific immune cells and immune responses.
- BCP-GNC regulates innate and adaptive immune responses in two stages (directing the M2 polarization of macrophages and inhibiting the maturation of dendritic cells) to promote osteoinduction.

**Electronic supplementary material:**

The online version of this article (https://doi.org/10.1007/s40820-020-00540-z) contains supplementary material, which is available to authorized users.

## Graphical abstract

**Figure:**
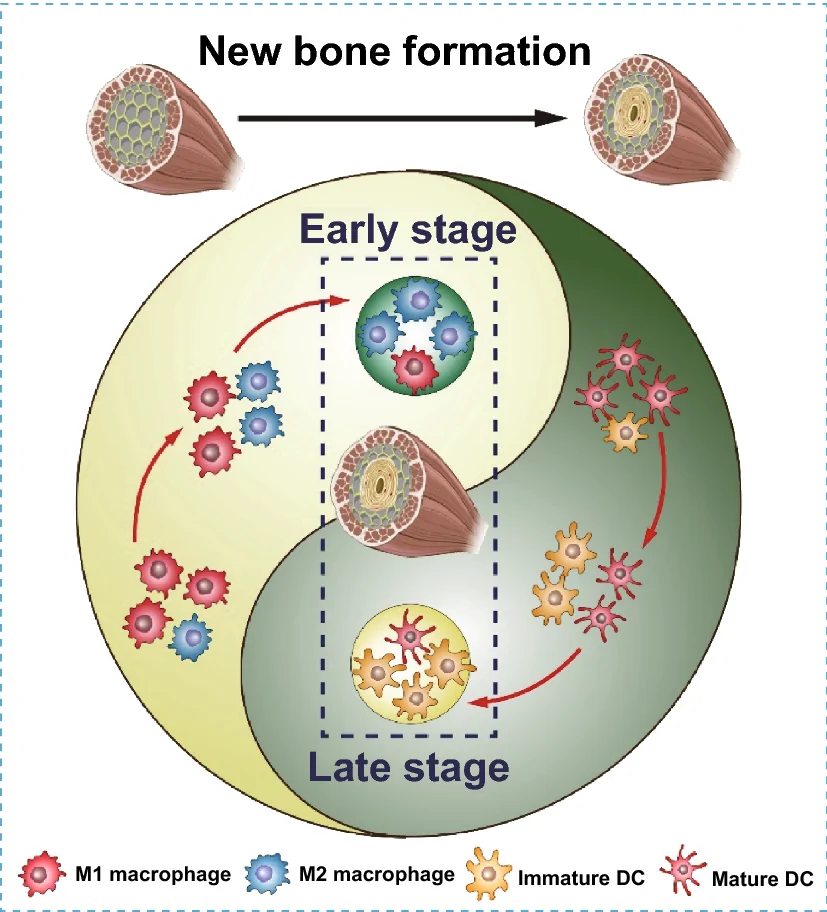

## Introduction

Osteoimmunomodulation is an important concept in the development and evaluation of bone biomaterials. An ideal bone biomaterial should be able to control the osteoimmune responses, resulting in the optimal inflammatory environment for stem cell recruitment and angiogenesis during bone regeneration. Therefore, osteoimmunomodulation provides a valuable strategy for the development of advanced bone biomaterials [1–4]. For example, T cells and natural killer cells can enhance the function of osteoclasts by releasing receptor activator of nuclear factor-κ B ligand (RANKL), thereby promoting inflammatory bone resorption [5, 6]. M2-polarized macrophage and regulatory T (Treg) cell subsets release anti-inflammatory cytokines and growth factors to reduce inflammation and promote the function of osteoblasts [3, 7]. Therefore, developing an in-depth understanding of the mechanism of osteoimmunomodulation during bone regeneration is of great significance for the development of advanced bone biomaterials.

The immune response of a biomaterial includes an early innate immune response and subsequent adaptive immune response. Macrophages are recruited into the implant region and participate in early innate immune response. To regulate the variable microenvironment after the implantation of a foreign body and subsequent injury repair, macrophages are transformed into a classical M1 or M2 polarization phenotype in a series of signal transductions, which are, respectively, mediated by inflammation and tissue homeostasis [4, 8]. This variable macrophage polarization is an important factor in tissue regeneration, with M2-type polarization being a favorable response to tissue regeneration [9, 10]. The surface shape [11, 12], nano-modification [13] and drug release [14] of biomaterials have been reported to regulate macrophage polarization and make the microenvironment more conducive to tissue regeneration. Dendritic cells (DCs), which are the most potent antigen-presenting cells, serve as bridges connecting innate and adaptive immunity by integrating and processing innate immune signals and relaying them to T cells [15, 16]. Therefore, the long-term immune response of a biomaterial is mainly determined by DCs and their ability to initiate and regulate the adaptive immune response. Specifically, the danger signals or foreign body immunogenicity caused by the implanted material induce DC maturation, resulting in the increased expression of DC costimulatory molecules (CD40, CD83 and CD86) and major histocompatibility complex class II (MHC II) [17]. The interaction of mature DCs (mDCs) with T cells activates the longer-term adaptive immune response. However, how biomaterials regulate DC maturation and the effects of adaptive immunity on bone induction remain unclear. In summary, macrophages and DCs play important roles in the osteoinductive processes of biomaterials by affecting multiple physiological stages, including the innate immune response, adaptive immune response and regeneration. Therefore, understanding the specific roles of macrophages and DCs in the osteogenic process and considering these roles in material development will improve our ability to control the early inflammatory response during bone regeneration.

Many materials for controlled drug release have been developed with a focus on controlling the immune responses involved in damage repair and anti-infection activity [14, 18–20]. Photosensitive delivery systems can stimulate beneficial photochemical reactions or release active molecules, thereby affecting the metabolism of osteoblasts [21–23]. This strategy has broad prospects in bone regeneration. Near-infrared (NIR) light can penetrate through tissue to depths reaching centimeters [24]. Moreover, controlling the transmission power of the NIR light can further improve its penetration depth [25]. This technique has valuable applications in, for example, the switching of implanted devices, photodynamic therapy, preoperative imaging and diagnostic imaging [24–26]. However, it remains challenging to accurately regulate the two stages of immune response (innate and adaptive) to promote the osteoinduction of biomaterials. In this study, we first assessed the essential roles of macrophages and DCs during biphasic calcium phosphate (BCP)-induced ectopic bone formation. We found that the osteoinductive capacity of BCP depends on the M2 macrophages and immature DCs (imDCs) within the implanted environment. Based on these findings, we designed a dual-targeting nano-in-micro scaffold to control the activation of macrophages and DCs via the controlled release of cell-specific regulators [interleukin-4 (IL-4) for macrophages and dexamethasone (DXMS) for DCs] through dual-wavelength excited gold nanocages (GNCs). The resulting BCP-GNC scaffold promotes the local enrichment of M2 macrophages and imDCs, which is beneficial for controlling local inflammation during new bone formation. The findings provide insights into osteoimmunomodulation during biomaterial-induced bone formation. This study is the first to develop an advanced bone biomaterial with specific immune cell targeting to regulate the early inflammatory response during osteogenesis.

## Experimental Section

### Ethical Approval and Mice Model

The treatment of experimental mice was carried out in strict accordance with the policy of Ethics Committee for Animal Research, Shandong First Medical University & Shandong Academy of Medical Sciences, China; as well as Wuhan University, China. The Ethics Committee for Animal Use approved it under protocol number 69/2017. CD11c-DTR, mice were C57BL/6 background and purchased from Jackson Laboratories. Mice were maintained in specific pathogen-free condition. The female C57BL/6 mice (6–8 weeks) were purchased from Vitalriver (Beijing, China). Mice implanted with biomaterials were tested by flow cytometry at 2 weeks and histological staining was performed at 4 days, 7 days, 4 weeks and 8 weeks.

### Depletion of DCs or Macrophages in vivo

For depletion of DCs in vivo, an injection (i.t.) of diphtheria toxin (DT, 100 ng per mice, Sigma, USA) was managed in CD11c-DTR mice or WT mice. Clodronate liposomes were prepared according to the method [27]. Briefly, 1% of the clodronate was encapsulated in the liposomes and 4 mL clodronate–liposome suspension contained 20 mg clodronate. In this study, for depletion of macrophages, an injection (i.v.) with of clodronate liposomes per mice (i.e., 50 mg kg^−1^) or control liposomes loaded equivalent PBS.

### Preparation of Single Cell Suspensions

Muscle was finely minced in RPMI-1640 containing collagenase type II (2 mg mL^−1^, Thermo Fisher Scientific Inc., USA) and type IV (1 mg mL^−1^). They were incubated for 1 h (37 °C 5% CO_2_). The remaining cell slurry was filtered through a 70 μm cell strainer and was centrifuged at 2500 rpm 4 °C 5 min. Discard the supernatant and resuspend the cells.

### Flow Cytometry

Cells were incubated with antibodies 4 °C 20 min. Cells were examined by Aria II Flow Cytometer (BD Bioscience, USA). Cells were gated as follows: DCs (F4/80^−^ CD11c^+^ IA/IE^+^), macrophages (CD11b^+^ F4/80^+^) and M1 macrophages (CD11b^+^ F4/80^+^ CD206^−^ MHC II^high^), M2 macrophages (CD11b^+^ F4/80^+^ CD206^+^ MHC II^low^). Intracellular cytokine staining: 2 µL mL^−1^ Cell Activation Cocktail (with Brefeldin A) (Cat: 423,303, Biolegend, USA) was used to incubate cells at 37 °C in a CO_2_ incubator for 6 h. Then, the Fixation/Permeabilization Solution Kit (Cat: 554,714, BD Biosciences, USA) was used to stimulus cells. After cell fixation and permeabilization (fixation/permeabilization solution, 100 uL/10^6^ cells, 4 °C, 30 min), the BD Perm/Wash™ Buffer is used to wash the cells and to dilute the IFN-γ antibody for staining (4 °C, 30 min). Antibodies: The following were purchased from BioLegend: anti-IA/IE (1:3200, Clone: M5/114.15.2, Cat: 107,630), anti-CD206 (1:200, Clone: C068C2, Cat: 141,705), anti-Ki67 (1:200, Clone: 16A8, Cat: 652,403), anti-IFN-γ (1:100, Clone: XMG1.2, Cat: 505,805). The following were purchased from eBioscience: anti-CD11b (1:200, Clone: M1/70, Cat: 47–0112-82), anti-CD11c (1:200, Clone: N418, Cat: 45–0114-82), anti-F4/80 (1:200, Clone: BM8, Cat: 17–4801-82).

### Histological Staining

The samples were used for the preparation of paraffin sections and those samples were immersed in 10% EDTA solution that was changed each day for 3 weeks in total for suitable decalcification. The gradient was then dehydrated with different concentrations of alcohol and embedding in paraffin. We performed haematoxylin–eosin (H&E), Masson, immunohistochemical (IHC) and immunofluorescent (IF) staining according to the protocol of the manufacturer (MXB biotechnologies, China). The primary antibodies were listed as: CD11c (1:200 for IF and 1:350 for IHC, Cat: 97,585, CST, USA), F4/80 (1:250, Cat: 70,076, CST, USA), Col1a1(1:100, Cat: SA2005, Boster, China), Runx2 (1:200, Cat: ab76956, Abcam, USA), CD146 (1:100, Cat: A13927, ABclonal, USA), CD83 (1:100, Cat: A2040, ABclonal, USA), CD40 (1:100, Cat: A0218, ABclonal, USA), iNOS (1:100, Sant Cruz, USA.), Arg1 (1:150, Cat: A11925, ABclonal, USA), CD3 (1:100, Cat: PB0112, Boster, China). For IF staining, the anti-mouse, goat and rabbit secondary antibodies with red and green fluorescent markers were bought from Abbkine (USA), respectively. The DAPI dye (Zhongshan Biotechnology, Ltd, China) stained nucleus of cells in tissue and cells. For the IHC staining, the 3,3-diaminobenzidine tetrahydrochloride (Zhongshan Biotechnology, Ltd, China) was used to visualized color development. The images of all stained sections were captured with an Olympus DP72 microscope (Olympus Corporation, Japanese). For the calculation method of osteogenic area and positive cells expression cells in the images, we selected five random repetitions around the biomaterials, finally analyzed the number of osteogenic areas or positive cells with these five repetitions.

### Cell Culture and Osteogenic Induction

RAW264.7 and DC2.4 cells were routinely cultured in DMEM (10% fetal bovine serum) at 37 °C with 5% CO_2_. Osteogenic inducing medium was composed of *α*-MEM, 10% FBS, 100 U mL^−1^ penicillin, 100 µg/mL streptomycin, *β*-glycerophosphate (10 mM), L-ascorbic acid (50 μg mL^−1^) and dexamethasone (10 nM). 1 × 10^6^ cells of RAW264.7 or DC2.4 were cultured with 2.5 mg scaffolds for 24 h and then the relevant tests were performed.

### Alizarin Red (AR) S Staining

It was carried out to evaluate the mineralization level of extracellular matrix. PBS were used to washed twice, fixed in 4% formaldehyde for 10 min at 26 °C, then stained with 0.1% Alizarin red S staining solution (1 h 37 °C pH = 4.2) and stop the reaction with distilled water three times. Finally, the images were taken by microstructures under optical microscope.

### RNA Extraction and RT-qPCR

In the light of the protocol of the manufacturer, extracted total RNA with Trizol reagent (TriPure Isolation Reagent, Roche Applied Science, Germany). The concentration of the total RNA was measured by Nanodrop2000 equipment (Thermo Fisher Scientific Inc., USA). The synthesized cDNA and RT-qPCR using PrimeScript RT-PCR Kit (TaKaRa, Japan) with reference to the protocol of TaKaRa. The primer sequences of target genes are shown in Table S1.

### Characterization and Synthesis of GNCs

As described in our previous study, GNCs were then synthesized utilizing a modified galvanic replacement reaction system [28, 29]. By measuring the ultraviolet (UV)-visible absorption spectrum of the sample extracted from these solutions, the degree of substitution can be lightly monitored until the absorption peaks are approximately 690 and 808 nm. The surface of GNCs was conjugated with thermally responsive 1-tetradecanol, whose conformation can be changed under the temperature variations. The different localized surface plasmon resonance (LSPR) peaks increase the surface temperature of the 690 or 808 nm GNCs. When heated above the low critical solution temperature (LCST) of 1-tetradecanol, the pores on the GNCs were exposed, causing the drug to release from the interiors [30, 31]. The 690 and 808 nm GNCs were maintained at 4 °C Then, the IL-4 (Cat: 214–14-50, PeproTech, USA) or DXMS (Cat: 1042-1G, Biovision, USA) was loaded into the GNCs through the phase change method (IL-4: 690 nm GNCs; DXMS: 808 nm GNCs) [28]. The GNCs carrying IL-4 or DXMS were then obtained through centrifugation. The GNCs were preserved in 4 °C for further use. Scanning electron microscope (SEM, Hitachi, Japan) was used to show the morphology of GNCs. The hydrodynamic diameters and the zeta potentials of both groups were examined by dynamic light scattering at room temperature. The UV–visible absorption spectra were recorded by a dual beam spectrophotometer (TU-1901, China) with the wavelength range from 400 to 900 nm at room temperature.

### Far-red and NIR Triggered Drug Release

To detect the drug release of 690 and 808 nm GNCs, we use fluorescein methylene blue (MB) as a model drug to load into GNCs. 690 nm far-red or 808 nm NIR controlled release of load model fluorescein MB. 1 mL 690 or 808 nm GNCs was exposed to irradiation for 4 min, 4 times repeatedly. The power density of irradiation was 1.0 W cm^−2^. The fluorescein MB released was detected with a fluorescence spectrometer to accurately monitor the release kinetics.

Before the two kinds of GNCs were irradiated together with 690 nm far-red and 808 nm NIR, we used Rhodamine B as another model drug to load into 808 nm GNCs. 1 mL 690 or 808 nm GNCs was exposed to irradiation for 15 min. The power density of irradiation was 1.0 W cm^−2^.

### Photothermal Behavior of GNCs

A concentration of 20 μg mL^−1^ 690 or 808 nm GNCs was measured under the wavelength of 690 and 808 nm, in which the different power is 1.0, 2.0 and 3.0 W cm^−2^. In addition, PBS was used as a control. In vivo, the hind limbs of mice were shaved; 0.1 mL suspension of 690 or 808 nm GNCs was injected into the deep part of the gastrocnemius muscle of mouse, which was consistent with the depth of biomaterials’ implantation. The 690 and 808 nm diode laser system (BWT Beijing Ltd, Beijing, China) were used to irradiated (1.0 W cm^−2^ power density) on the surface of injected site. At the same time, the FLIR A65sc Test Kit (FLIR Systems, Inc. USA) was used for thermal imaging recording and temperature detection.

### In Vitro Cytotoxicity Experiments

RAW264.7 were employed to investigate the biocompatibility of the BCP-GNCs. Cells were co-cultured with the GNCs solutions in different concentrations (0, 5, 10, 20, 50 and 100 μg mL^−1^) for about 72 h. Then, cell counting kit (CCK)-8 was added and the samples were kept 1.5 h in the incubator. Cell viability was assessed by measuring the absorbance at 450 nm using a micro-plate reader (Spectra Max M2, MDC, USA).

### Characterization and Synthesis of BCP-GNCs

The ceramics were synthesized by the wet chemical precipitation method [32]. The BCP ceramics were made with a HA/beta-tricalcium phosphate (*β*-TCP) ratio of 60/40 through sintering at a temperature of 1100 °C. The 690 nm GNCs loaded with IL-4 and 808 nm GNCs loaded with DXMS were incubated with BCP for 12 h at 4 °C. Finally, air-dry at room temperature to obtain BCP-GNCs. Each 2.5 mg of BCP is combined with 600 ng 690 nm GNCs and 600 ng 808 nm GNCs. Scanning electron microscope (SEM, SU7000, Hitachi, Japan) was used to show the morphology of BCP and BCP-GNCs.

### Model of Skeletal Muscle Implant Material in vivo

Mice were anesthetized by intraperitoneal injection of 1% sodium pentobarbital and then disinfected in the surgical field. The epidermis and muscle were cut out from the outside of the soleus muscle of the hind leg of the mouse and the wound was unified by 1 cm; 2.5 mg of materials (BCP, *β*-TCP or BCP-GNCs) was implanted in the middle third of the gastrocnemius muscle bundle and the wound was sutured. In the mice implanted with BCP-GNCs, 690 nm light was used to stimulate the release of IL-4 at 1, 2 and 3 days after implantation; 808 nm light was used to stimulate the release of DXMS at 4, 5 and 6 days after implantation. Light was irradiated on the surface of mice at the surgical site, centered on the implanted BCP-GNCs, with an area of 1 cm^2^, a power of 1.0 W and each time of 3 min. The 690 and 808 nm diode laser system were used for the above solutions. When taking the material, the model mice were first euthanized by carbon dioxide treatment and then the middle third of the soleus muscle was taken for tissue embedding or single cell preparation.

### Statistical Analysis

All experiments in vitro were repeated three times and there were at least three replicates in each group. In all animal experiments, female mice aged 6–8 weeks were randomly selected as the corresponding treatment group. Each group consisted of 5–6 mice and performed 2–3 independent experiments. The data were expressed as the mean ± standard deviation. The semi-quantification data were tested by Bartlett’s and Kolmogorov–Smirnov tests for homogeneity and normal distribution. Significant differences among each group were evaluated by One-way ANOVA and a post hoc *t* test. *P* < 0.05 were considered as statistical significance. All statistical analyses were done through the GraphPad Prism software 7.0 (GraphPad, San Diego, CA, USA).

## Results and Discussion

### Mesenchymal Stem Cell (MSC) Recruitment and Osteoblast Differentiation in New Bone Formation with BCP

Calcium phosphate (CaP) materials are similar in composition to the inorganic component of bone. Due to their great bioactivity, biocompatibility and osteoconductivity, CaP materials including hydroxyapatite (HA), alpha- and beta-tricalcium phosphate (*α*-TCP and *β*-TCP, respectively) and BCP are clinically used as graft materials in bone bioengineering [33]. However, these materials lack osteoinductivity unless osteogenic agents are added to the graft material before implantation [34, 35]. Among these CaP materials, *β*-TCP is composed of a single *β*-TCP phase, whereas BCP comprises HA and *β*-TCP in a 60/40 ratio. In BCP, HA is the more stable phase, while *β*-TCP is the more soluble phase [36, 37]. Due to the diphasic composition of BCP, we speculated that it would exhibit better stability as a scaffold compared to *β*-TCP while also providing sufficient degradation to provide space for the growth of cells and proteins [36, 38]. Therefore, we expect BCP to show more active bone regeneration than *β*-TCP alone. Other factors, including surface topography, porosity and particle size, may also affect the performance of bone graft materials and were considered in this study [39, 40].

**Fig. 1:**
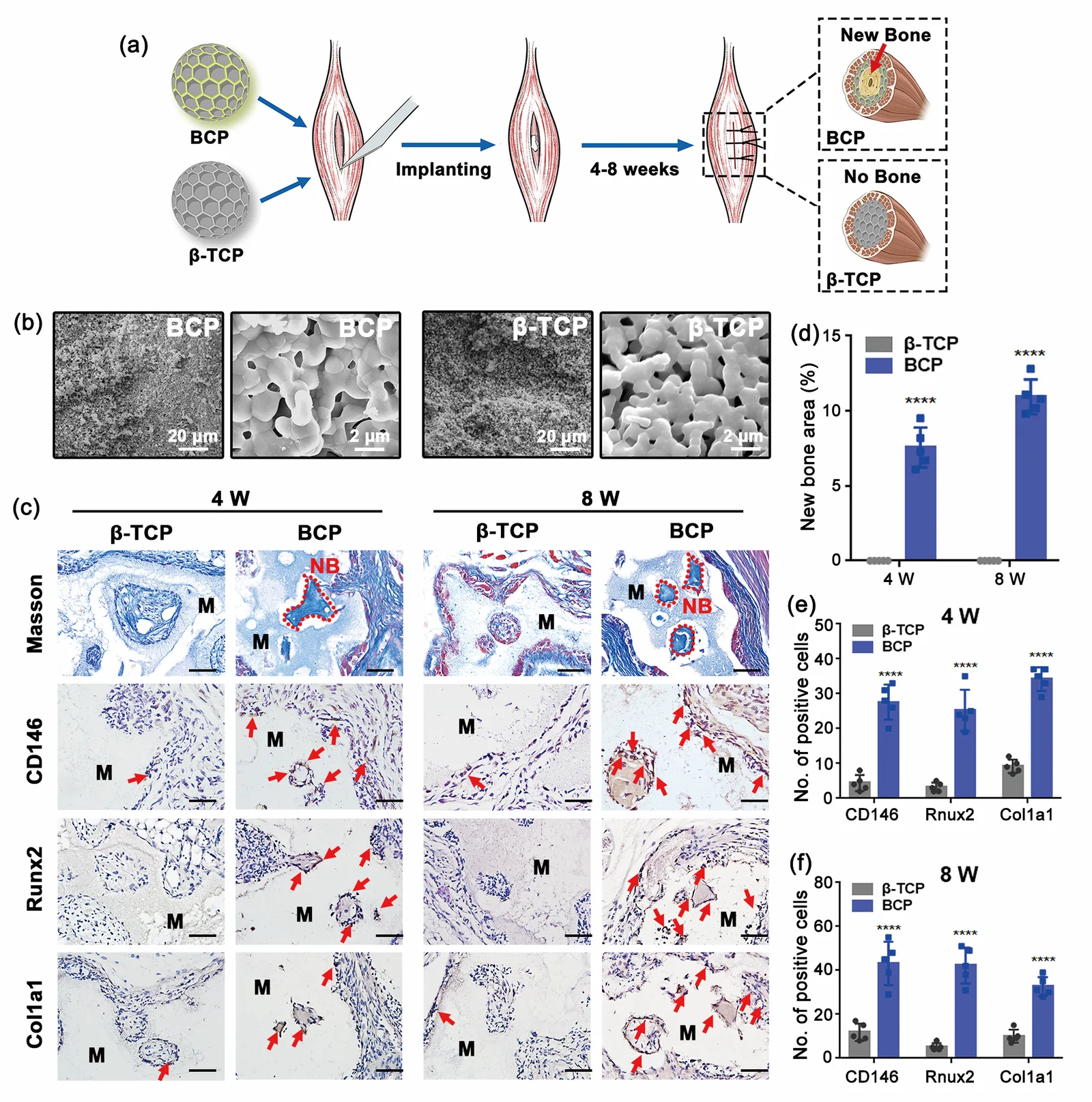
**a** Schematic diagram of BCP and *β*-TCP implantation and new bone formation. **b** Scheme, morphology and nanostructure of BCP and *β*-TCP observed by SEM. **c** Masson staining and IHC staining (CD146, Runx2 and Col1a1: MSC and osteoblast markers, red arrow) of implant area of BCP and *β*-TCP in vivo after 4 weeks (4 W) and 8 weeks (8 W) (the red dash line shows the new bone formation area; NB, new bone; M, material. Scale bar = 100 μm). **d**–**f** Semiquantification of new bone area and positively stained cells in (**d**). n = 5, ****P < 0.0001

Currently, ideal bone induction biomaterials are considered to exhibit three key characteristics [41]. First, they should be able to recruit MSCs. Second, they should be able to convert undifferentiated MSCs into mature osteoblasts. Finally, they should be able to induce endogenous heterotopic bone formation in tissues other than bone. In this study, we compared the ectopic bone formation abilities of BCP and β-TCP in mouse skeletal muscle at four and eight weeks after implantation (Fig. 1a). X-ray diffraction (XRD) analysis confirmed that the two materials were indeed BCP and β-TCP (Fig. S1). Whether implanted in vivo or cultured in vitro, both BCP and β-TCP were particles. The scanning electron microscopy (SEM) images show that BCP and β-TCP possessed interlaced micro-structured surfaces (1–2 μm) with epitaxial polygonal surface structures and crater-like holes (Figs. 1b and S2). To investigate the abilities of the two scaffolds to promote ectopic bone formation, we implanted BCP and *β*-TCP into mouse skeletal muscle without loading cytokines. Four weeks after implantation, Masson staining and hematoxylin and eosin (H&E) staining revealed significant new bone formation in the BCP-implanted area. In contrast, no obvious new bone was found around the *β*-TCP-implanted area, even at eight weeks after implantation (Figs. 1c, d and S3). In this study, the osteogenic area of BCP ceramic was the centers of the pores of BCP rather than the pore walls, as is typically the case [42]. This may be related to the surface porosity and degradation of the BCP synthesized in this study. Figure S2 shows that our BCP ceramic had low porosity and shallow pores. These shallow pores required further degradation to form deeper pores that facilitate the penetration and growth of cells and molecules. Therefore, the new bone was formed in the centers of the pores. In addition, histological staining indicated that the new bone was immature bone or osteoid. This may be related to the porosity, particle size and/or surface topography of BCP, which all play key roles in its osteoinduction [40].

Although Masson staining and H&E staining indicated that the new bone area was immature bone or osteoid, the markers of MSCs and osteoblasts were detected by immunohistochemistry (IHC) staining. The key factors determining the osteoinductive effects of biomaterials are the recruitment and osteogenic differentiation of host MSCs [41]. When the tissue is pathologically damaged, resident MSCs, many from the bone marrow, will be recruited to the injured area and then differentiate into osteoblasts for bone regeneration. In this process, osteoinductive biomaterials provide spaces for MSC growth and absorb endogenous growth factors to promote the recruitment and osteogenic differentiation of MSCs [41]. Here, IHC staining demonstrated that the recruited cells obviously expressed CD146 (the surface markers for MSCs) along with Runx2 and Col1a1 (markers for osteoblasts) in the implant area at 4–8 weeks after implantation in the BCP group (Figs. 1c, e and f). In summary, compared to *β*-TCP, BCP had a stronger ability to promote the osteogenic differentiation of MSCs, consistent with our previous findings [43].

Increasing evidence [2–4, 44] suggests that the immune microenvironment can affect the behavior of MSCs. Osteoimmunomodulation theory suggests that the interactions between immune cells and MSCs affect new bone formation. In addition, immune response occurs before the bone formation process, indicating that immune cells are involved in the regulation of biomaterials. Therefore, investigating the immunomodulatory effects of osteoinductive materials is a promising direction to uncover the mechanism of biomaterial-induced bone formation.

### BCP-Induced MSC Osteogenic Differentiation by Promoting M2 Macrophage Polarization

**Fig. 2:**
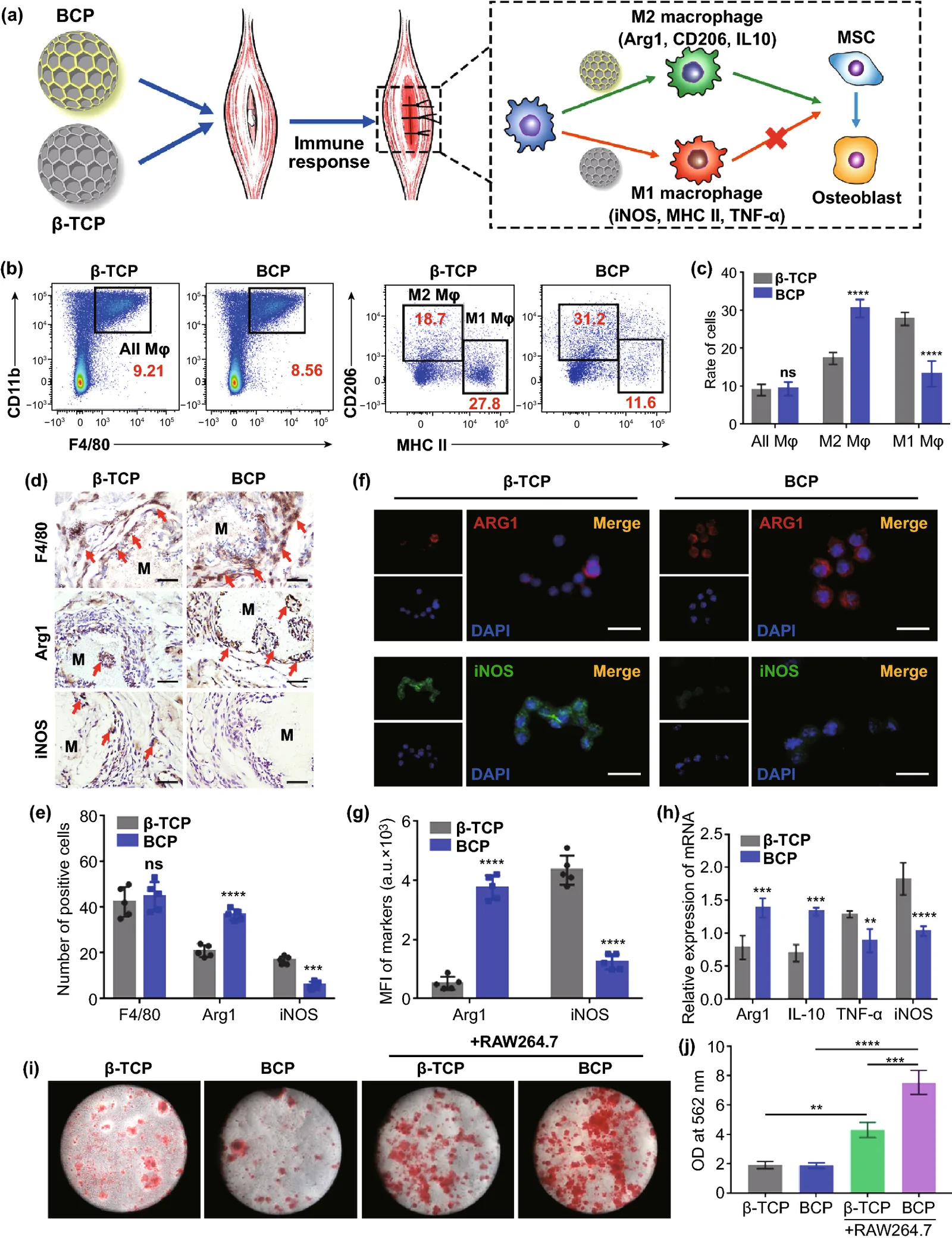
**a** Schematic diagram of biomaterial-mediated macrophage polarization and MSC osteogenesis. **b** FACS of all macrophages (F4/80 + , CD11b +), M2 macrophages (F4/80 + , CD11b + , CD206^high^, MHC II^low^) and M1 macrophages (F4/80 + , CD11b + , CD206^low^, MHC II^high^) recruitment at 2 weeks after biomaterials implantation in vivo. **c** Semiquantification of gating strategy cells in (B). n = 6, ****P < 0.0001, ns: not significant. **d** IHC staining of F4/80—macrophage marker, Arg1—M2 macrophage marker and iNOS—M1 macrophage marker at 4 weeks after biomaterials implantation in vivo. Red arrow, positive cell. Scale bar = 100 μm. M, material. **e** Semiquantification of positively stained cells in (**d**). n = 5, ***P < 0.001, ****P < 0.0001, ns: not significant. **f** Immunofluorescent (IF) staining of Arg1 (red), iNOS (green) and nuclei (blue) after macrophages seeded on the BCP or *β*-TCP for 24 h. Scale bar = 5 μm. **g** Semiquantification of positively stained cells in (**f**). n = 5, ****P < 0.0001. **h** Relative mRNA expressions of macrophage polarization-related genes. M2 macrophage polarization-related anti-inflammatory genes (Arg1, IL-10) and the M1 macrophage-related pro-inflammatory gene (TNF-α, iNOS). n = 3, **P < 0.01, ***P < 0.001, ****P < 0.0001. **i, j** ARS staining of MSCs on day 21 and semiquantification of mineralized nodules. n = 3, **P < 0.01, ***P < 0.001, ****P < 0.0001

Macrophages, which are the most important innate immune cells, are regulated by damage signals or foreign body immunogens. In addition to playing an important role in the initial natural immune response, macrophages also play a key regulatory role in the tissue repair phase. Moreover, the type of macrophage polarization during the innate immune response determines the roles of the macrophages in tissue repair [8–10], indicating that macrophage polarization during the innate immune response is critical for the regulation of osteogenesis. Recent studies have shown that macrophages undergo two major types of polarization: M1 polarization promotes inflammation, while M2 polarization induces tissue regeneration [8–10]. M1 macrophages can release inflammatory factors, including tumor necrosis factor *α* (TNF-*α)*, IL-6 and IL-1*β*, which contribute to tissue inflammation and osteoclast formation and are unhelpful for bone formation. In contrast, M2 macrophages can produce transforming growth factor *β* (TGF-*β*), IL-10 and arginase-1 (Arg1), which are involved in tissue repair and can promote osteogenesis. To explore the role of macrophages in the process of biomaterial osteoinduction, macrophage polarization and MSC osteogenic differentiation were detected after BCP and *β*-TCP scaffolds were implanted into mouse skeletal muscle (Fig. 2a). Two weeks after implantation, the total macrophages (F4/80 + and CD11b +) around the two scaffolds, as determined by flow cytometry (FCM), were not significantly different. However, the proportion of M1 macrophages (CD206^−^ and MHC II^high^) in the BCP group was significantly lower than in the *β*-TCP group, while the proportion of M2 macrophages (CD206 + and MHC II^low^) was two times higher (Fig. 2b, c). The FCM gating strategy used in this study is shown in Fig. S4. IHC staining of the scaffolds and surrounding areas also indicated that the total macrophages (F4/80 +) did not differ significantly between the groups at four weeks after implantation. The expression of M2 macrophage marker (Arg1 +) was two times higher in the BCP group than in the *β*-TCP group, while the expression of M1 macrophage marker (iNOS +) was mostly attenuated (Fig. 2d, e). The immunofluorescence staining of macrophages co-cultured with BCP or *β*-TCP in vitro confirmed the above findings (Fig. 2f, g). Under BCP stimulation, higher expressions of M2 polarization-related mRNA (Arg1 and IL-10) and lower expressions of M1 polarization-related mRNA (TNF-*α* and iNOS) were found compared to *β*-TCP (Fig. 2h). These results confirm that compared to *β*-TCP, BCP had a better ability to promote the M2 polarization of macrophages and inhibit M1 polarization, which inhibits inflammation and promotes bone formation, consistent with our previous study [43]. Chen and colleagues also found that compared to *β*-TCP, BCP upregulated the expression of M2 macrophage marker CD206 in vitro and increased the number of Arg1 + M2 macrophages in vivo [42]. These and our results both suggest that BCP can promote the M2 polarization of macrophages to a greater extent than *β*-TCP. The development of M1 macrophages and long-term inflammation could lead to abnormal tissue repair. However, the initial inflammatory and M1 macrophages may also promote the recruitment of MSCs and vascular progenitor cells during tissue regeneration. This program is activated by C–C motif chemokine ligand 2 (CCL2), CXC chemokine ligand 8 (CXCL8) and stromal cell-derived factor 1 (SDF-1), which are secreted by activated M1 macrophages [45].

To further investigate whether BCP can promote MSC differentiation into osteoblasts through M2 macrophages, we co-cultured macrophages with BCP or *β*-TCP for 24 h and then collected the supernatants for incubation with MSCs. MSCs with the leachate alone were used as a control. After 14 days of incubation, the cells were stained with alizarin red and the osteogenic differentiation of the MSCs was assessed based on the morphologies of the deeply stained mineralized nodules. The mRNA expressions of the osteogenic genes alkaline phosphatase (ALP), osteocalcin (OCN), runt-related transcription factor 2 (Runx2) and osterix (Osx) were detected by real-time quantitative polymerase chain reaction (RT-qPCR). Based on the alizarin red staining results, the BCP + RAW264.7 group had more mineralized nodules than other groups, while no difference in mineralized nodules was observed between the BCP and *β*-TCP control groups that were not co-cultured with RAW264.7 cells (Fig. 2i, j). The RT-qPCR analysis showed high levels of mRNA expression in the osteogenic genes ALP, OCN, Runx2 and Osx in the BCP + RAW264.7 group (Fig. S5). These findings further demonstrate that BCP promoted the M2 polarization of macrophages, which in turn promoted MSC osteogenesis. This may be due to the secretion of some anti-inflammatory factors and growth factors (e.g., IL-10 and TGF-β) from M2 macrophages, which positively affect stem cell recruitment and differentiation [46]. In conclusion, the results of both in vivo and in vitro studies demonstrated that BCP can activate M2 macrophage polarization, thereby promoting the osteogenic differentiation and mineralization of MSCs and subsequently enhancing new bone formation.

### BCP Reduced T Cell Proliferation and Activation by Inhibiting DC Maturation

**Fig. 3:**
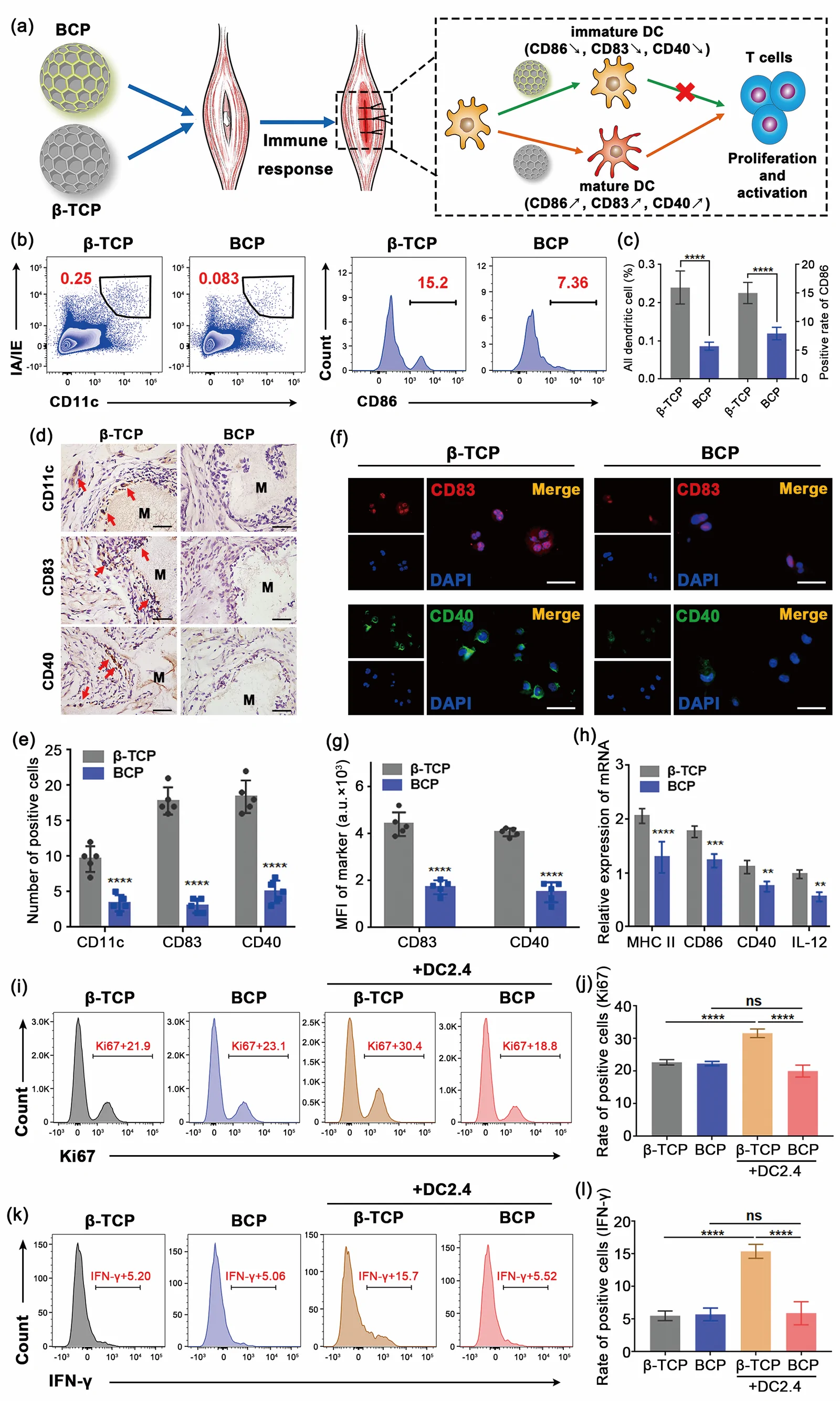
**a** Schematic diagram of biomaterial-mediated DC maturation and T cell activation. **b** FACS of all DCs (CD11c + , IA/IE +) and mature DCs (CD11c + , IA/IE + , CD86 +) recruitment at 2 weeks after biomaterials implantation in vivo. **c** Semiquantification of gating strategy cells in (B). n = 6, ****P < 0.0001. **d** IHC staining of CD11c—DC marker and CD83/CD40—mature DC markers at 4 weeks after biomaterials implantation in vivo. Red arrow, positive cell. Scale bar = 100 μm. M, material. **e** Semiquantification of positively stained cells in (**d**). n = 5, ****P < 0.0001.** f** Immunofluorescent (IF) staining of CD83 (red), CD40 (green) and nuclei (blue) after DCs seeded on the BCP or *β*-TCP for 24 h. Scale bar = 5 μm. **g** Semiquantification of positively stained cells in (**f**). n = 5, ****P < 0.0001, ns: not significant. **h** Relative mRNA expressions of DC maturation genes (MHC II, CD86, CD40, IL-12). n = 3, **P < 0.01, ***P < 0.001, ****P < 0.0001. **i-l** FACS of T cell proliferation (Ki67 +) and activation (IFN-γ) and semiquantification of positive cell rate. n = 6, ***P < 0.001, ****P < 0.0001, ns: not significant

In addition to innate immunity, adaptive immunity is also important in tissue regeneration. Although initially viewed as having a secondary role in tissue regeneration, the adaptive immune response was recently reported to play a crucial role in tissue repair and regeneration, particularly with respect to T cell activity [47–49]. One possible explanation for this is that the adaptive immune response overlaps temporally and spatially with MSC recruitment and differentiation, which generally occur in the timeframe of one week to months after biomaterials implantation and are co-located in the damaged area [50]. This spatial and temporal overlap ensures the interaction of adaptive immune cells (especially T cells) with stem cells. Researchers in the fields of regenerative medicine and materials science believe that an excessive and prolonged adaptive immune response (excessive T cell activation and proliferation) is detrimental to tissue regeneration and reduces the biocompatibility of biomaterials, leading to failed regeneration or foreign body rejection [50]. Notably, as a bridge between innate and adaptive immunity, DCs play a dominant role in adaptive immune response. When a biomaterial contacts blood or interstitial fluid, proteins and other macromolecules directly adsorb onto the biomaterial surface. These proteins may be foreign substances or autoantigens. Regardless of the source, they can directly activate DCs or be processed and expressed by DCs, thereby triggering an adaptive immune response [50]. These stimuli include the deposition of complement 3 (C3) on the biomaterial surface and the release of soluble danger signals during the death of necrotic cells caused by injury (e.g., DNA, RNA and HMGB1) [51]. To explore the role of DCs in the adaptive immune response of implanted biomaterials, DC maturation along with T cell proliferation and activation were evaluated after BCP and *β*-TCP were implanted into mouse skeletal muscle (Fig. 3a). At two weeks after implantation, FCM analysis showed that the BCP-implanted area contained three times fewer DCs (CD11c + and IA/IE +) than the *β*-TCP-implanted area and the degree of DCs maturation (CD86) was significantly lower in the BCP group than in the *β*-TCP group (Fig. 3b, c).

The IHC staining of the scaffolds and surrounding areas indicated fewer mDCs (CD11c + , CD83 +  and CD40 +) around BCP compared to around *β*-TCP at four weeks after implantation (Fig. 3d, e). This finding is consistent with the immunofluorescence staining results of DC2.4 cells co-cultured with BCP and *β*-TCP in vitro (Fig. 3f, g). Under BCP stimulation, the expressions of mDC-related mRNA (MHC II, CD86, CD40 and IL-12) were restrained compared to *β*-TCP (Fig. 3h). These results confirm that BCP inhibits DC maturation both in vivo and in vitro. DCs can be regulated by the physicochemical properties of biomaterials through an immune recognition/sensing mechanism involving multiple toll-like receptor (TLR)/myeloid differentiation factor 88 (MyD88)-dependent signaling pathways (particularly TLR2, TLR4 and TLR6). The interaction of TLRs with biomaterials can induce the expression of mDC markers and pro-inflammatory factors (e.g., IL-1*β*, IL-6, TNF-*α* and IL-12), which gives DCs the ability to activate T cells [52].

To further investigate whether BCP affects T cell responses through DC maturation, we used the two scaffolds to stimulate co-cultured systems of DCs and T cells for 24 h. We then collected the mixed cells to label T cells (CD3 +) along with their proliferation marker (Ki67) and activation marker (IFN-γ). FCM was used to detect the differences in T cell proliferation and activation. T cells with scaffold supernatant alone (no DCs) were used as the control. The results show that T cells without DCs exhibited lower proliferation ability and activation status, with no difference observed between the two biomaterials. In the presence of DCs, the *β*-TCP group showed stronger T cell proliferation and activation than the BCP group (Fig. 3i–l). IHC staining after 4 weeks (4 W) implantation revealed fewer CD3 + T cells around BCP than around *β*-TCP (Fig. S6). These results indicate that BCP reduced the proliferation and activation of T cells by inhibiting the maturation of DCs. Thus, BCP controlled the microenvironment to induce a low-intensity adaptive immune response and provide a mild immune microenvironment for new bone formation. These findings are in agreement with reports that T cell subsets inhibit bone regeneration during the fracture healing process. For example, in Rag1^−/−^ mice (mouse models without functional T cells or B cells), fracture healing accelerated [49] and effector memory CD8 + T cells secrete IFN-*γ* and TNF-*α* to delay bone formation and lengthen the fracture healing process [47]. In conclusion, imDCs play a key role in BCP osteoinduction by inhibiting T cell proliferation and activation to provide an adaptive immune environment that facilitates osteogenesis.

### Design and Characterization of the Dual-targeting Nano-in-micro Scaffold (BCP-GNC)

**Fig. 4:**
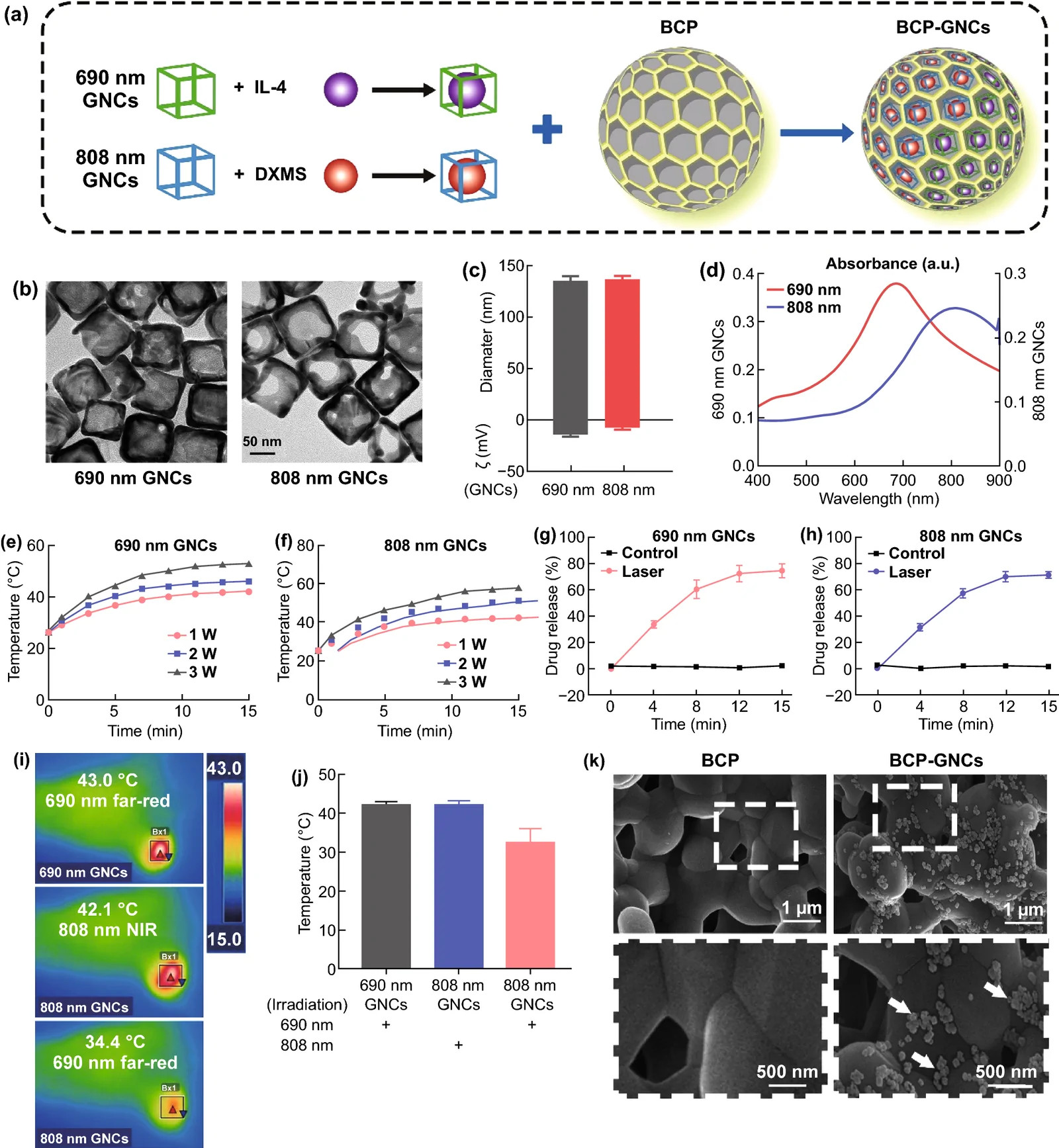
**a** Schematic diagram of synthetic process of BCP-GNCs. **b** TEM images of 690 nm GNCs and 808 nm GNCs (bar = 50 nm). **c** DLS measurements of 690 nm GNCs and 808 nm GNCs hydrodynamic size (diameter) and zeta potential (ζ). n = 3. **d** Absorbance spectra of 690 nm GNCs and 808 nm GNCs. **e, f** Temperature increase of 690 nm GNCs and 808 nm GNCs under irradiation (690 nm far-red or 808 nm NIR) at varying power densities (1 W, 2 W, 3 W). **g, h** The fluorescein methylene blue (MB) was used as a model drug to load into GNCs. 690 nm far-red or 808 nm NIR controlled release of fluorescein MB load in 690 nm GNCs and 808 nm GNCs. **i** Infrared thermal images of 690 nm GNCs and 808 nm GNCs under the 690 nm far-red and 808 nm NIR in vivo. **j** Semiquantification of the temperature in infrared thermal images in (**i**). **k** SEM images of GNCs compositing BCP (bar = 500 nm)

Based on the above results, we designed a nanosystem for controlled drug release that targets macrophages and DCs to improve the osteoinductive effect of biomaterials by modifying the innate immune and adaptive immune environment. The designed system features dual-wavelength photosensitive drug release to both promote macrophage M2 polarization and inhibit DC maturation. We selected IL-4 (a classic inducer of M2 macrophage polarization) [14] to be released in the innate immune phase and DXMS (an effective inducer of imDCs or tolerogenic DCs) [53] for release in the later stage to inhibit DC maturation and T cell activation. As shown in Fig. 4a, the dual-wavelength photosensitive GNCs (690 nm GNCs and 808 nm GNCs) were synthesized via current displacement reaction using a silver nano-copper template. IL-4 was then loaded in the 690 nm GNCs, while DXMS was loaded into the 808 nm GNCs. Finally, the two drug-loaded GNCs were assembled with the BCP scaffold to create the dual-targeting nano-in-micro scaffold (i.e., BCP-GNC). The transmission electron microscopy images of the GNCs in Fig. 4b show the porous and hollow nanostructures of both GNCs. Dynamic light scattering (DLS) measurements indicated that the hydrodynamic diameters of the 690 and 808 nm GNCs were approximately 135 nm. The surface zeta potential of the 690 nm GNCs was more negative than that of the 808 nm GNCs (Fig. 4c). GNCs have shown good biocompatibility, which is a fundamental criteria for a drug delivery platform [54]. When used in vivo, the GNCs will remain in the body for a certain time before and after the drug is released. The biocompatibility of the GNCs in this study was evaluated by CCK-8 assay. Cell viability was not significantly affected when the GNC concentration was lower than 20 μg mL^−1^; however, at concentrations at or above 50 μg mL^−1^, cell death reached approximately 40% (Fig. S7). The ultraviolet–visible (UV–Vis) absorption spectra confirmed the characteristic peaks of the GNCs at 690 and 808 nm (Fig. 4d), indicating that the GNCs should release the two drugs independently at the two wavelengths. To verify the photothermal effect of the dual-wavelength photosensitive GNCs, the temperature was controlled by varying the powers of the 690 nm (far-red) and 808 nm (NIR) sources from 1 to 3 W cm^−2^. The temperature of the 690 nm GNCs under 690 nm far-red excitation increased from 25 to 48 °C (Fig. 4e), while that of the 808 nm GNCs under 808 nm NIR excitation increased from 25 to 58 °C (Fig. 4f). The drug loading efficiencies in the 690 and 808 nm GNCs were approximately 9.12% and 10.25%, respectively, while the encapsulation efficiencies were approximately 73.59% and 70.82%, respectively. The photo-controlled release curves of the two drug-loaded GNCs are similar to the photothermal curves (Fig. 4g, h), demonstrating the desired photosensitive drug release. To demonstrate the dual drug-release of 690 and 808 nm GNCs, we irradiated the two kinds of GNCs together with 690 nm far-red for 15 min first and then with 808 nm NIR for 15 min later. The photo-controlled release curves shown that 690 nm far-red light only has a significant controlled release effect on 690 nm GNCs in the first 15 min and has a weak effect on 808 nm GNCs. However, 808 nm NIR obviously stimulated the drug release of 808 nm GNCs in the last 15 min (Fig. S8). To realize the sequential release of IL-4 from the 690 nm GNCs followed by the release of DXMS from the 808 nm GNCs, we clarified whether the photothermal effect of the 808 nm GNCs could be activated under 690 nm far-red irradiation in vivo. As expected, the temperature of the 808 nm GNCs irradiated with 690 nm far-red light only reached 34.4 °C, much lower than that of the control groups (43.0 °C for the 690 nm GNCs irradiated with 690 nm far-red light and 42.1 °C for the 808 nm GNCs irradiated with 808 nm NIR light; Figs. 4i, j). Thus, 690 nm far-red irradiation had little effect on the 808 nm GNCs. In Fig. 4i, the experiments were done in mouse. The 690 or 808 nm GNCs was implanted in the muscle. Hair removal was performed on the body surface corresponding to the implantation area of GNCs to avoid the interference of hair on the thermal imager. Although animal experiments in mice are close to practical applications, there are still differences in external temperature, surface temperature and deep tissue temperature in mouse. Therefore, the experiments performed at a constant 37 °C would be expected to be realized in the future. Finally, the two successfully validated GNCs were assembled with the BCP scaffold. The SEM images of BCP-GNC demonstrate the successful loading of the GNCs onto the BCP scaffold (Fig. 4k). After immersion in simulated body fluid (SBF) for 7 d, the stability of BCP-GNC was again confirmed by SEM. The results indicate that a large amount of GNCs remained on the surface or inside of BCP after immersion in SBF (Fig. S9). Thus, BCP-GNC was successfully constructed for subsequent studies. A photograph of BCP before loading with GNCs is also shown in Fig. S10.

### Controlled Release of IL-4 and DXMS Improves New Bone Formation by Promoting M2 Macrophages and imDCs

**Fig. 5:**
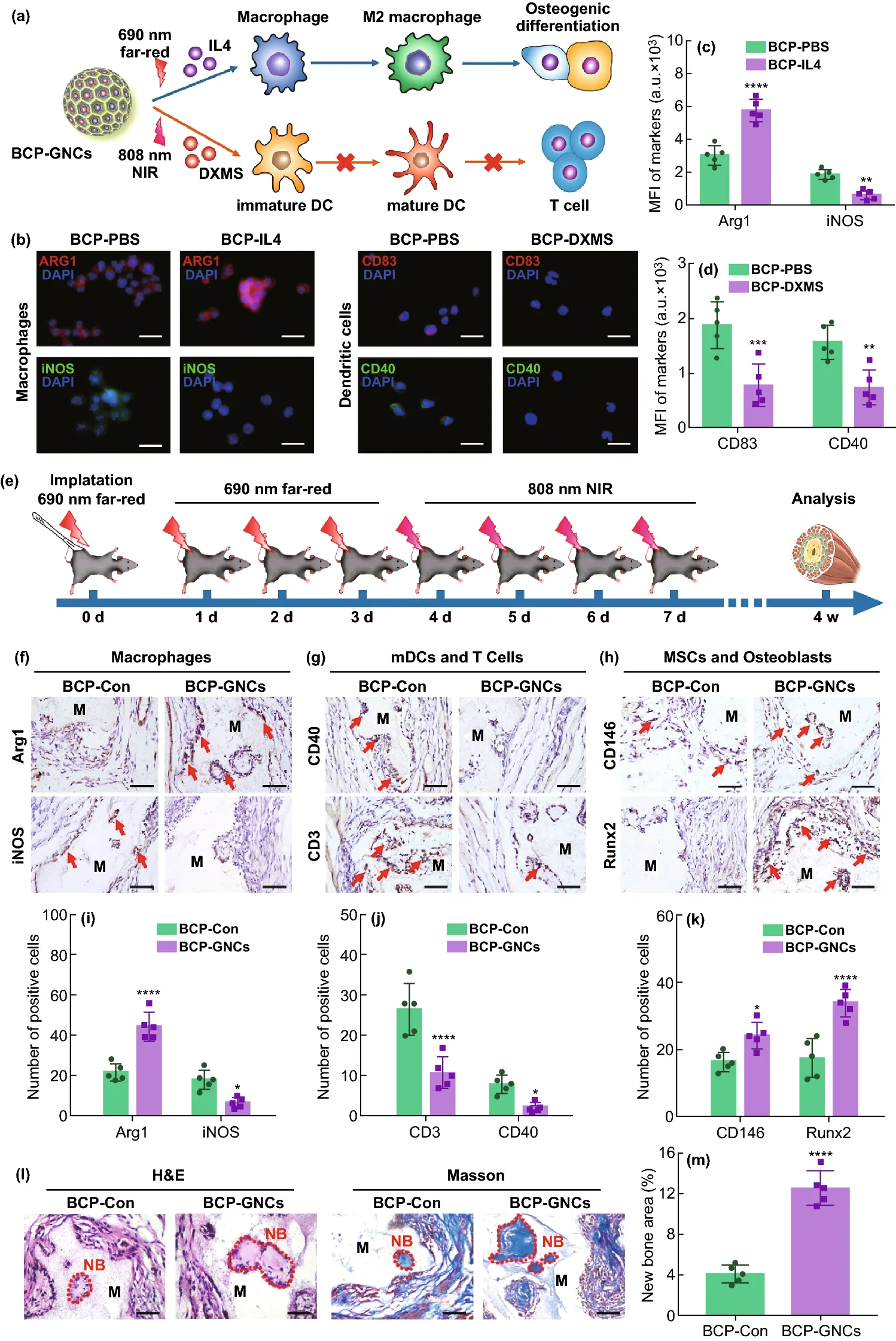
**a** Schematic diagram of BCP-GNCs-mediated macrophage polarization and DC maturation by releasing IL-4 and DXMS. **b** IF staining of Arg1 (red) and iNOS (green) of macrophage, CD83 (red) and CD40 (green) of DC and nuclei (blue) after macrophages or DCs seeded on the BCP with 690 nm far-red or 808 nm NIR stimulating the release of PBS (BCP-PBS), 690 nm far-red stimulating the release of IL-4 (BCP-IL4) or 808 nm NIR stimulating the release of DXMS (BCP-DXMS) for 24 h. Scale bar = 5 μm. **c, d** Semiquantification of positively stained cells in (**b**). n = 5, **P < 0.01, ***P < 0.001. **e** Flowchart of time course for irradiating BCP-GNC in vivo. **f–h** IHC staining of M1, M2 macrophages (iNOS and Arg1), mature DCs (CD40), T cells (CD3), MSCs and osteoblasts (CD146, Runx2) under the BCP-GNCs implant in vivo. Scale bar = 100 μm. Red arrow, positive cells. M, material. **i**–**k** Semiquantification of positively stained cells in (E, F, G). n = 5, *P < 0.05, **P < 0.01, ****P < 0.0001. **l** H&E and Masson staining of implant area of BCP-Con and BCP-GNCs in vivo after 4 weeks (4 W) (the red dash line shows the new bone formation area; NB, new bone; M, material). **m** Semiquantification of new bone area in (**k**). n = 5, ****P < 0.0001

In order to visually detect the dual controlled release effect of 690 and 808 nm GNC, we used two fluorescein alternative drugs in the initial experiment. Although this method can dynamically monitor the dual release of drugs through the wavelength range of fluorescence, fluorescein alternative drugs are not actual therapeutic drugs and may have deviations in accuracy. Therefore, we further explored the biological effects of IL-4 and DXMS in vitro and in vivo. To validate the effects of BCP-GNC on macrophages and DCs, we investigated macrophage polarization under IL-4 stimulation and DC maturation under DXMS stimulation. The IL-4 was released from the BCP-GNC by 690 nm far-red and the DXMS was released by 808 nm NIR (Fig. 5a). In vitro studies revealed that IL-4-released BCP-GNC favors the M2 polarization of macrophages (red-Arg1) over the M1 polarization (green-iNOS). DXMS-released BCP-GNC decreased the expressions of mDC markers (CD83 and CD40; Fig. 5b–d). This confirmed that IL-4 and DXMS can successfully induce M2 macrophage polarization and reduce the maturation of dendritic cells in the BCP-stimulated immune cell environment.

We then used the ectopic bone formation model to investigate the effects of BCP-GNC on immune regulation and osteogenesis in vivo*.* Before application, we evaluated the biosafety of photothermal treatment since excessive temperatures may cause irreversible damage to tissues. First, we recorded the extreme temperatures generated by irradiation at different powers (1.0 and 2.0 W cm^−2^). At the power of 1 W cm^−2^, the maximum temperatures induced by 690 nm far-red and 808 nm NIR irradiation were approximately 42.0 and 43.1 °C, respectively; at 2.0 W cm^−2^, the corresponding maximum temperatures were approximately 46.2 and 50.3 °C, respectively. According to the recommended temperature for conventional photothermal therapy, the overall temperature of the tissue should be maintained between 42 and 45 °C. If the temperature exceeds 45 °C, the nearby normal tissue and drugs will be damaged through heat transfer, causing side effects and inhibiting the therapeutic effect [55]. In particular, IL-4, which was loaded in the 690 nm GNCs in this study, is likely to lose activity at high temperature. Thus, we selected 1.0 W cm^−2^ as the in vivo irradiation power to control the maximum temperature of the 690 nm GNCs to around 42 °C, although the activity of IL-4 may decrease slightly at this temperature. To achieve the immune regulation and osteogenesis effects of BCP-GNCs in vivo after implantation, BCP-GNCs were set to release IL-4 (targeting macrophages) during the innate immune response phase (0–3 days after implantation) followed by DXMS (targeting DCs) during the DC-activate T cells phase (4–7 days after implantation) [50]. After the BCP-GNCs implanted into the mouse, the time course of irradiations were executed following the flowchart (Fig. 5e). In vitro experiments (Fig. 4g, h), 690 and 808 nm GNC have reached the limit of drug release after 12 min irradiation. Continuing irradiation for a period of time did not significantly increase their drug release. In Fig. 5e, 690 nm far-red and 808 nm NIR were performed 4 times in vivo. To ensure sufficient drug release for 4 times, we determined that the time of each irradiation is 3 min in vivo. At 4 weeks after implantation, IHC staining showed stronger expressions of total macrophage marker (CD68), M2 macrophage marker (Arg1), MSC marker (CD146) and osteoblast markers (Runx2 and Col1a1) around BCP-GNC as well as lower expressions of M1 macrophage marker (iNOS), DC activation markers (CD83 and CD40) and T cell marker (CD3; Figs. 5f–k and S11–S13). Considering that the release times of IL-4 and DXMS by BCP-GNCs were much earlier than 4 weeks, we performed the polarization of macrophages after the release of IL-4 (at day 4) and the maturation of DCs after the release of DXMS (at day 7). The IHC staining for macrophages showed that Arg1 was high expression in BCP-GNC group at day 4 (Fig. S14). For DCs, CD40 was low expression in BCP-GNC group at day 7 (Fig. S15). These results benefit to clarify the effect of dual drug release (IL-4 and DXMS) on M2 macrophages and imDCs. Moreover, H&E and Masson staining showed more new bone formation in the BCP-GNCs group than the BCP-Con group (Fig. 5l, m). In conclusion, there is an important effect of osteoimmunomodulation on osteoinduction during biomaterials implantation. BCP-GNC improved the local immune microenvironment through the controlled release of two drugs, resulting in stronger M2 polarization and weaker DC maturation to promote new bone formation.

### Impairing New Bone Formation by Macrophage Depletion and Promoting DC Depletion

**Fig. 6:**
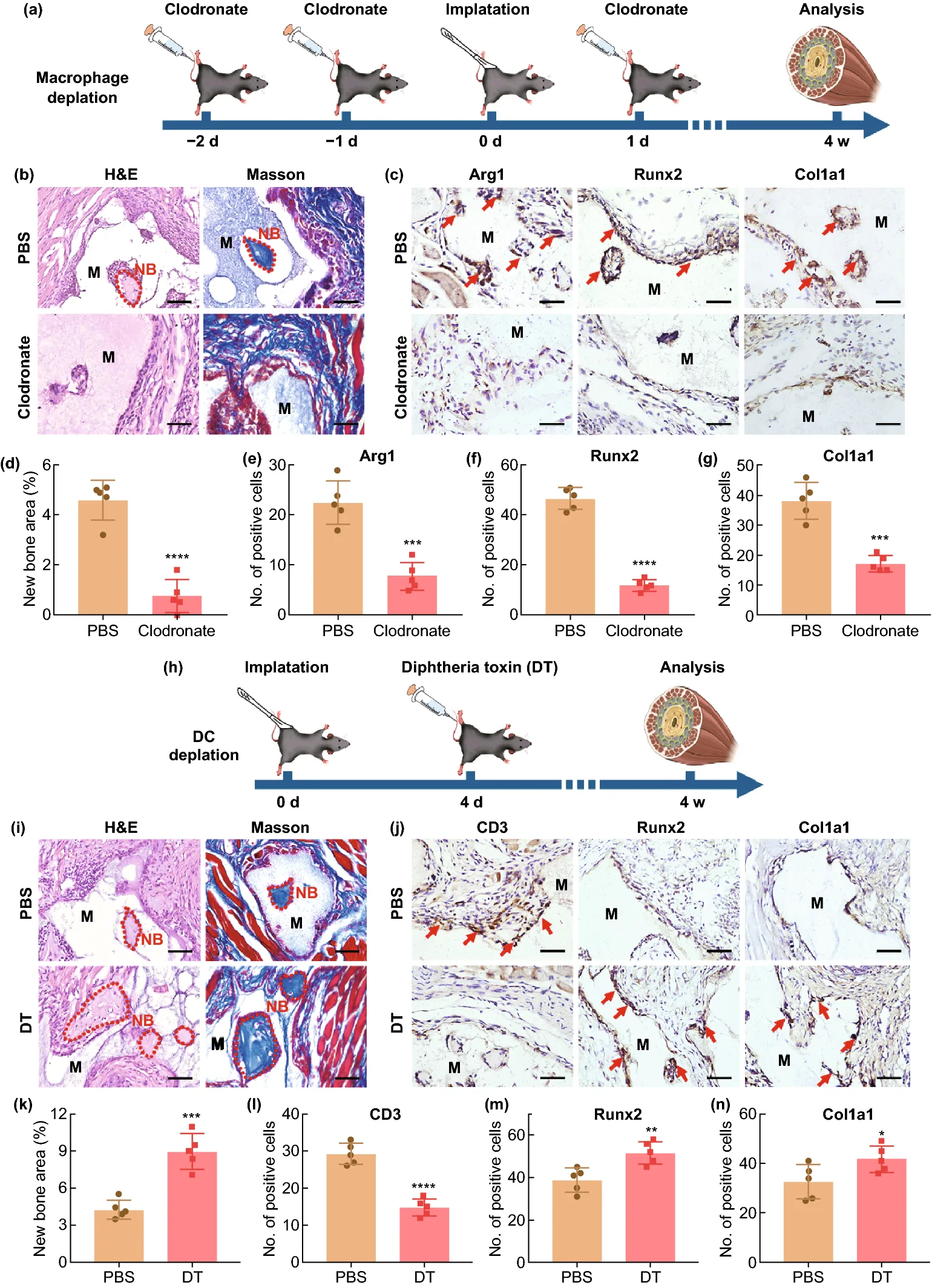
**a** Implementation strategy of clodronate on macrophages depletion. **b** H&E and Masson staining of BCP implant area treating with PBS or clodronate in vivo after implant 4 weeks (the red dash line shows the new bone formation area; NB, new bone; M, material). Scale bar = 100 μm. **c** IHC staining of M2 macrophages (Arg1) and osteoblasts (Runx2, Col1a1) under the BCP implant treating with PBS or clodronate in vivo. Scale bar = 100 μm. Red arrow, positive cells. M, material. **d**–**g** Semiquantification of new bone area and positively stained cells in (**b, c**). n = 5, ***P < 0.001, ****P < 0.0001. **h** Implementation strategy of diphtheria toxin (DT) on DCs depletion. **i** H&E and Masson staining of BCP implant area treating with PBS or DT in vivo after implant 4 weeks (the red dash line shows the new bone formation area; NB, new bone; M, material). Scale bar = 100 μm. **j** IHC staining of T cells (CD3) and osteoblasts (Runx2, Col1a1) under the BCP implant treating with PBS or DT in vivo. Scale bar = 100 μm. Red arrow, positive cells. M, material. **k**–**n** Semiquantification of new bone area and positively stained cells in (**i, j**). n = 5, *P < 0.05, **P < 0.01, ***P < 0.001, ****P < 0.0001

To further confirm the important roles of macrophages and DCs in the osteoinduction of biomaterials, we depleted mice mononuclear/macrophages and DCs through clodronate liposome injection and the use of CD11c-DTR mice models, respectively. To deplete early macrophages, which play an important role in the early-stage innate immune response, clodronate liposomes were injected two days (− 2 d) prior to the implantation of BCP and lasted until one day (1 d) after implantation (Fig. 6a). The H&E and Masson staining of tissues surrounding BCP at four weeks after implantation revealed that the depletion of macrophages inhibited the heterotopic ossification ability of BCP. IHC staining showed that the expressions of M2 macrophage marker (Arg1) and osteoblast markers (Runx2 and Col1a1) were significantly attenuated in the macrophage depletion group than PBS group (Fig. 6b–g). This was supported by previous reports that macrophage depletion inhibited the recruitment of MSCs and reduced MSC-mediated tissue regeneration [46]. In summary, the lack of macrophages during immune response can impair biomaterial-mediated osteogenesis. It is worth noting that clodronate liposomes deplete all phagocytes, including monocytes, neutrophils and mast cells, in addition to macrophages. These phagocytes also participate in the early inflammation responses of biomaterials. In addition, the effect of clodronate on osteoclasts may directly affect the bone regeneration of biomaterials. Although this scheme for depleting macrophages in vivo may create unpredictable interfering effects, the validity of studying macrophages using this scheme is widely recognized [56–58].

To deplete DCs during the important stage of DC-active T cells (days 4–7), DT was injected into CD11c-DTR mice on the third day after BCP implantation (Fig. 6h). At four weeks after implantation, the H&E and Masson staining of tissues surrounding BCP revealed that the depletion of DCs promoted heterotopic ossification. IHC staining showed that the expressions of T cell markers (CD3) and osteoblast markers (Runx2 and Col1a1) were significantly enhanced in the DC-depleted group than PBS group (Fig. 6i–n). Previous studies found that the depletion of DCs impeded T cell activation and adaptive immune response, which reduced inflammation and provided a facilitative microenvironment for bone regeneration [59]. Another study found that imDC induced the differentiation of native T cells into Treg cells, which secreted large amounts of TGF-*t* to accelerate tissue regeneration [60, 61]. In summary, the lack of macrophages during immune response impaired biomaterial-mediated osteogenesis, whereas the lack of DCs promoted it.

Notably, the immune response after the implantation of a biological material includes both innate and adaptive immune responses but may not be limited to the responses of macrophages and DCs. Evidence suggests that the infiltration of neutrophils and monocytes around biomaterials occurs in the early stage after implantation and can be regulated by biomaterials to induce MSC recruitment, angiogenesis and bone regeneration [62].

## Conclusions

**Fig. 7:**
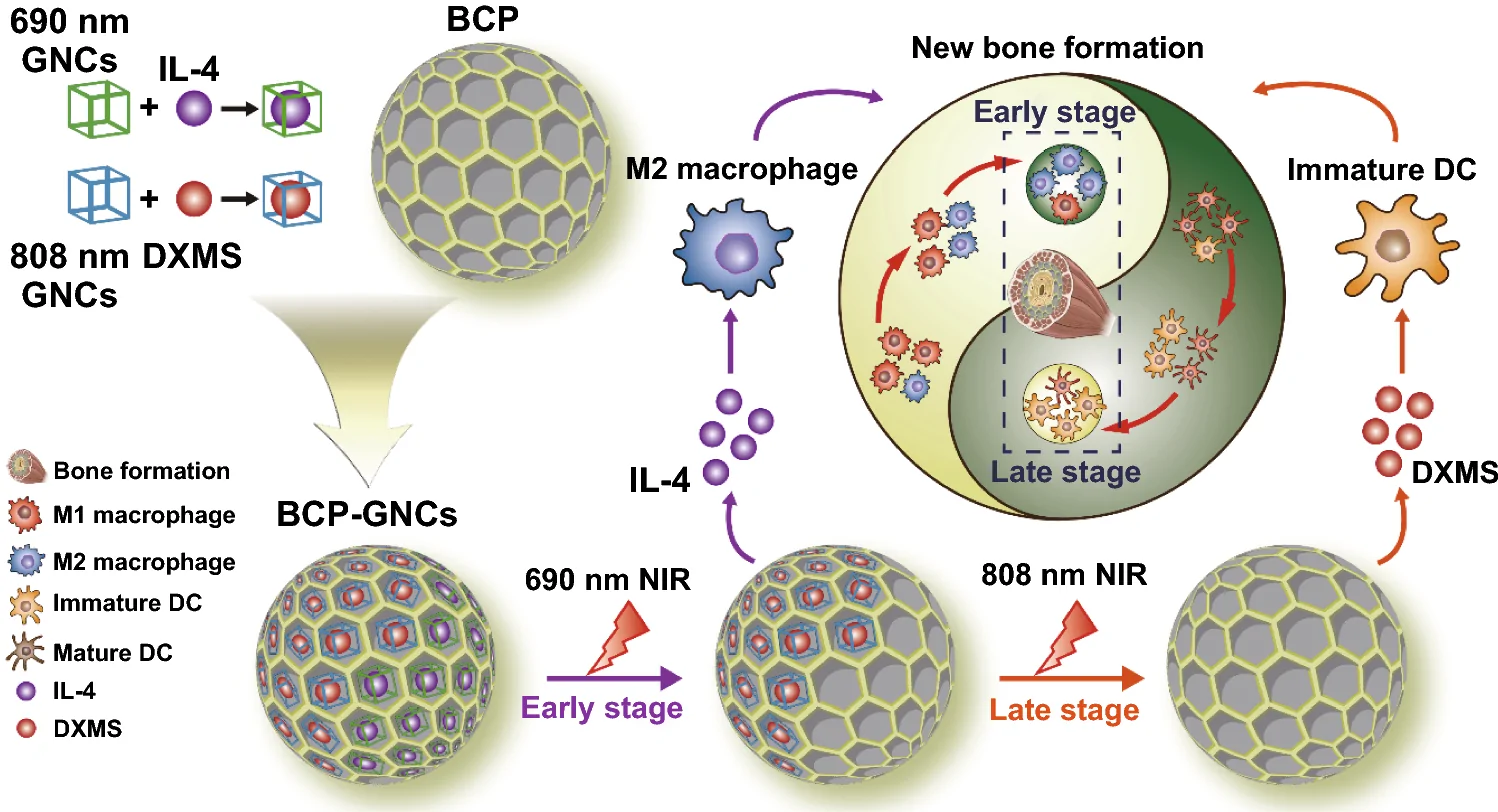
Gold nanocages (GNCs) modification of BCP according to the immune response characteristics of macrophages and DCs and controlled release of IL-4 at the early stage implantation and DXMS at the late stage facilitate macrophage M2 polarization and inhibition of DC maturation to promote materials-mediated new bone formation

This study elucidated the immunological mechanism by which BCP promotes new bone formation. We first demonstrated the dual role of BCP in osteoinduction; BCP both promotes M2 macrophage polarization and inhibits DC maturation, leading to enhanced MSC osteogenic differentiation and the inhibition of T cell activation. Considering the important roles of macrophages and DCs in the osteoinductive process of biomaterials in multiple physiological stages (during the innate immune response, adaptive immune response and bone regeneration), we designed BCP-GNC, a dual-targeting nano-in-micro scaffold, to modulate macrophages and DCs. BCP-GNC improved the local immune microenvironment through the controlled release of IL-4 and DXMS. BCP-GNC enhanced M2 polarization by releasing IL-4 in the early stage after implantation and inhibited DC maturation by releasing DXMS in the later stage, thereby promoting new bone formation (Fig. 7). The findings provide a new immunological approach to biomaterial-mediated bone regeneration along with a strategy to overcome challenges in bone tissue engineering by precisely regulating innate and adaptive immune responses.

## Electronic supplementary material

Below is the link to the electronic supplementary material.

Supplementary file1 (PDF 1654 kb)

## References

[1] W. Liu, J. Li, M. Cheng, Q. Wang, K.W.K. Yeung et al., Zinc-modified sulfonated polyetheretherketone surface with immunomodulatory function for guiding cell fate and bone regeneration. Adv. Sci. **5**(10), 1800749 (2018). 10.1002/advs.201800749 PMC6193167 30356934

[2] L. Bai, Z. Du, J. Du, W. Yao, J. Zhang et al., A multifaceted coating on titanium dictates osteoimmunomodulation and osteo/angio-genesis towards ameliorative osseointegration. Biomaterials **162**, 154–169 (2018). 10.1016/j.biomaterials.2018.02.010 29454274

[3] J.M. Sadowska, F. Wei, J. Guo, J. Guillem-Marti, M.P. Ginebra et al., Effect of nano-structural properties of biomimetic hydroxyapatite on osteoimmunomodulation. Biomaterials **181**, 318–332 (2018). 10.1016/j.biomaterials.2018.07.058 30098568

[4] K. Okamoto, T. Nakashima, M. Shinohara, T. Negishi-Koga, N. Komatsu et al., Osteoimmunology: The conceptual framework unifying the immune and skeletal systems. Physiol. Rev. **97**(4), 1295–1349 (2017). 10.1152/physrev.00036.2016 28814613

[5] H. Li, S. Hong, J. Qian, Y. Zheng, J. Yang et al., Cross talk between the bone and immune systems: Osteoclasts function as antigen-presenting cells and activate CD4+ and CD8+ T cells. Blood **116**(2), 210–217 (2010). 10.1182/blood-2009-11-255026 PMC2910608 20304810

[6] A.J. Ashcroft, S.M. Cruickshank, P.I. Croucher, M.J. Perry, S. Rollinson et al., Colonic dendritic cells, intestinal inflammation and t cell-mediated bone destruction are modulated by recombinant osteoprotegerin. Immunity **19**(6), 849–861 (2003). 10.1016/s1074-7613(03)00326-1 14670302

[7] A.M. Tyagi, M. Yu, T.M. Darby, C. Vaccaro, J.Y. Li et al., The microbial metabolite butyrate stimulates bone formation via T regulatory cell-mediated regulation of WNT10b expression. Immunity **49**(6), 1116–1131 (2018). 10.1016/j.immuni.2018.10.013 PMC6345170 30446387

[8] P.J. Murray, Macrophage polarization. Annu. Rev. Physiol. **79**, 541–566 (2017). 10.1146/annurev-physiol-022516-034339 27813830

[9] M.M. Alvarez, J.C. Liu, G. Trujillo-de Santiago, B.H. Cha, A. Vishwakarma et al., Delivery strategies to control inflammatory response Modulating M1–M2 polarization in tissue engineering applications. J Control Release. **240**, 349–363 (2016). 10.1016/j.jconrel.2016.01.026 PMC4945478 26778695

[10] K.L. Spiller, R.R. Anfang, K.J. Spiller, J. Ng, K.R. Nakazawa et al., The role of macrophage phenotype in vascularization of tissue engineering scaffolds. Biomaterials **35**(15), 4477–4488 (2014). 10.1016/j.biomaterials.2014.02.012 PMC4000280 24589361

[11] Z. Chen, A. Bachhuka, S. Han, F. Wei, S. Lu et al., Tuning chemistry and topography of nanoengineered surfaces to manipulate immune response for bone regeneration applications. ACS Nano **11**(5), 4494–4506 (2017). 10.1021/acsnano.6b07808 28414902

[12] M. Tavakolian, S.M. Jafari, T.G.M.van de Ven, , A review on surface-functionalized cellulosic nanostructures as biocompatible antibacterial materials. Nano-Micro Lett. **12**, 73 (2020). 10.1007/s40820-020-0408-4 PMC7770792 34138290

[13] L. Bai, Y. Liu, Z. Du, Z. Weng, W. Yao et al., Differential effect of hydroxyapatite nano-particle versus nano-rod decorated titanium micro-surface on osseointegration. Acta Biomater. **76**, 344–358 (2018). 10.1016/j.actbio.2018.06.023 29908975

[14] D. Kwon, B.G. Cha, Y. Cho, J. Min, E.B. Park et al., Extra-large pore mesoporous silica nanoparticles for directing in vivo M2 macrophage polarization by delivering Il-4. Nano Lett. **17**(5), 2747–2756 (2017). 10.1021/acs.nanolett.6b04130 28422506

[15] D. Esterhazy, J. Loschko, M. London, V. Jove, T.Y. Oliveira et al., Classical dendritic cells are required for dietary antigen-mediated induction of peripheral T(REG) cells and tolerance. Nat. Immunol. **17**(5), 545–555 (2016). 10.1038/ni.3408 PMC4837106 27019226

[16] B. Pulendran, H. Tang, S. Manicassamy, Programming dendritic cells to induce T(H)2 and tolerogenic responses. Nat. Immunol. **11**(8), 647–655 (2010). 10.1038/ni.1894 20644570

[17] J. Park, J.E. Babensee, Differential functional effects of biomaterials on dendritic cell maturation. Acta Biomater. **8**(10), 3606–3617 (2012). 10.1016/j.actbio.2012.06.006 PMC3970713 22705044

[18] J. He, G. Chen, M. Liu, Z. Xu, H. Chen et al., Scaffold strategies for modulating immune microenvironment during bone regeneration. Mater Sci Eng C Mater Biol Appl. **108**, 110411 (2020). 10.1016/j.msec.2019.110411 31923946

[19] S. Maher, A. Mazinani, M.R. Barati, D. Losic, Engineered titanium implants for localized drug delivery: Recent advances and perspectives of titania nanotubes arrays. Expert Opin. Drug Deliv. **15**(10), 1021–1037 (2018). 10.1080/17425247.2018.1517743 30259776

[20] S. Behzadi, G.A. Luther, M.B. Harris, O.C. Farokhzad, M. Mahmoudi, Nanomedicine for safe healing of bone trauma: Opportunities and challenges. Biomaterials **146**, 168–182 (2017). 10.1016/j.biomaterials.2017.09.005 PMC5706116 28918266

[21] C. Xu, L. Xiao, Y. Cao, Y. He, C. Lei et al., Mesoporous silica rods with cone shaped pores modulate inflammation and deliver BMP-2 for bone regeneration. Nano Res. **13**(9), 2323–2331 (2020). 10.1007/s12274-020-2783-z

[22] S. Hosseinpour, R. Fekrazad, P.R. Arany, Q. Ye, Molecular impacts of photobiomodulation on bone regeneration: A systematic review. Prog. Biophys. Mol. Biol. **149**, 147–159 (2019). 10.1016/j.pbiomolbio.2019.04.005 31002851

[23] B. Byambaa, N. Annabi, K. Yue, G. Trujillo-de Santiago, M.M. Alvarez et al., Bioprinted osteogenic and vasculogenic patterns for engineering 3d bone tissue. Adv Healthc Mater (2017). 10.1002/adhm.201700015 PMC11034848 28524375

[24] A.L. Vahrmeijer, M. Hutteman, J.R. van der Vorst, C.J. van de Velde, J.V. Frangioni, Image-guided cancer surgery using near-infrared fluorescence. Nat. Rev. Clin. Oncol. **10**(9), 507–518 (2013). 10.1038/nrclinonc.2013.123 PMC3755013 23881033

[25] Y.A. Nakamura, S. Okuyama, A. Furusawa, T. Nagaya, D. Fujimura et al., Near-infrared photoimmunotherapy through bone. Cancer Sci. **110**(12), 3689–3694 (2019). 10.1111/cas.14203 PMC6890452 31553485

[26] Y. Sun, H. Tu, S. You, C. Zhang, Y.Z. Liu et al., Detection of weak near-infrared optical imaging signals under ambient light by optical parametric amplification. Opt. Lett. **44**(17), 4391–4394 (2019). 10.1364/OL.44.004391 PMC7272329 31465409

[27] J. Hao, T. Han, M. Wang, Q. Zhuang, X. Wang et al., Temporary suppression the sequestrated function of host macrophages for better nanoparticles tumor delivery. Drug Deliv. **25**(1), 1289–1301 (2018). 10.1080/10717544.2018.1474965 PMC6058532 29869519

[28] C. Wang, Y. Wang, L. Zhang, R.J. Miron, J. Liang et al., Pretreated macrophage-membrane-coated gold nanocages for precise drug delivery for treatment of bacterial infections. Adv. Mater. **30**(46), e1804023 (2018). 10.1002/adma.201804023 30285289

[29] Q. Zhao, J. Wang, C. Yin, P. Zhang, J. Zhang et al., Near-infrared light-sensitive nano neuro-immune blocker capsule relieves pain and enhances the innate immune response for necrotizing infection. Nano Lett. **19**(9), 5904–5914 (2019). 10.1021/acs.nanolett.9b01459 31389707

[30] B. Pang, X. Yang, Y. Xia, Putting gold nanocages to work for optical imaging, controlled release and cancer theranostics. Nanomedicine **11**(13), 1715–1728 (2016). 10.2217/nnm-2016-0109 PMC5827786 27348546

[31] G.D. Moon, S.W. Choi, X. Cai, W. Li, E.C. Cho et al., A new theranostic system based on gold nanocages and phase-change materials with unique features for photoacoustic imaging and controlled release. J. Am. Chem. Soc. **133**(13), 4762–4765 (2011). 10.1021/ja200894u PMC3073071 21401092

[32] D. Xidaki, P. Agrafioti, D. Diomatari, A. Kaminari, E. Tsalavoutas-Psarras et al., Synthesis of hydroxyapatite, beta-tricalcium phosphate and biphasic calcium phosphate particles to act as local delivery carriers of curcumin: Loading, release and in vitro studies. Materials (2018). 10.3390/ma11040595 PMC5951479 29649121

[33] M. Ebrahimi, M.G. Botelho, S.V. Dorozhkin, Biphasic calcium phosphates bioceramics (ha/tcp): Concept, physicochemical properties and the impact of standardization of study protocols in biomaterials research. Mater Sci Eng C Mater Biol Appl. **71**, 1293–1312 (2017). 10.1016/j.msec.2016.11.039 27987685

[34] Y. Cai, X. Wang, C.K. Poh, H.C. Tan, M.T. Soe et al., Accelerated bone growth in vitro by the conjugation of BMP2 peptide with hydroxyapatite on titanium alloy. Colloids Surf. B Biointerfaces **116**, 681–686 (2014). 10.1016/j.colsurfb.2013.11.004 24304498

[35] M. Shi, R. Yang, Q. Li, K. Lv, R.J. Miron et al., Inorganic self-assembled bioactive artificial proto-osteocells inducing bone regeneration. ACS Appl. Mater. Interfaces **10**(13), 10718–10728 (2018). 10.1021/acsami.8b00385 29528210

[36] Y.M. Kong, H.E. Kim, H.W. Kim, Phase conversion of tricalcium phosphate into Ca-deficient apatite during sintering of hydroxyapatite-tricalcium phosphate biphasic ceramics. J Biomed Mater Res B Appl Biomater. **84**(2), 334–339 (2008). 10.1002/jbm.b.30876 17595029

[37] A. Fatimi, J.F. Tassin, M.A. Axelos, P. Weiss, The stability mechanisms of an injectable calcium phosphate ceramic suspension. J. Mater. Sci. Mater. Med. **21**(6), 1799–1809 (2010). 10.1007/s10856-010-4047-z PMC3118935 20229185

[38] M. Bohner, R.J. Miron, A proposed mechanism for material-induced heterotopic ossification. Mater. Today **22**, 132–141 (2019). 10.1016/j.mattod.2018.10.036

[39] M. Ebrahimi, P. Pripatnanont, N. Monmaturapoj, S. Suttapreyasri, Fabrication and characterization of novel nano hydroxyapatite/beta-tricalcium phosphate scaffolds in three different composition ratios. J. Biomed. Mater. Res. A **100**(9), 2260–2268 (2012). 10.1002/jbm.a.34160 22499354

[40] Y. Cai, L. Guo, H. Shen, X. An, H. Jiang et al., Degradability, bioactivity and osteogenesis of biocomposite scaffolds of lithium-containing mesoporous bioglass and MPEG-PLGA-b-PLL copolymer. Int. J. Nanomedicine **10**, 4125–4136 (2015). 10.2147/ijn.S82945 PMC4484672 26150718

[41] R.J. Miron, Y.F. Zhang, Osteoinduction: A review of old concepts with new standards. J. Dent. Res. **91**(8), 736–744 (2012). 10.1177/0022034511435260 22318372

[42] X. Chen, M. Wang, F. Chen, J. Wang, X. Li et al., Correlations between macrophage polarization and osteoinduction of porous calcium phosphate ceramics. Acta Biomater. **103**, 318–332 (2020). 10.1016/j.actbio.2019.12.019 31857257

[43] X. Jia, H. Xu, R.J. Miron, C. Yin, X. Zhang et al., EZH1 is associated with TCP-induced bone regeneration through macrophage polarization. Stem. Cells Int. **2018**, 6310560 (2018). 10.1155/2018/6310560 PMC6136473 30228822

[44] Z. Chen, R.M. Visalakshan, J. Guo, F. Wei, L. Zhang et al., Plasma deposited poly-oxazoline nanotextured surfaces dictate osteoimmunomodulation towards ameliorative osteogenesis. Acta Biomater. **96**, 568–581 (2019). 10.1016/j.actbio.2019.06.058 31271882

[45] J. Pajarinen, T. Lin, E. Gibon, Y. Kohno, M. Maruyama et al., Mesenchymal stem cell-macrophage crosstalk and bone healing. Biomaterials **196**, 80–89 (2019). 10.1016/j.biomaterials.2017.12.025 PMC6028312 29329642

[46] S.S. Jin, D.Q. He, D. Luo, Y. Wang, M. Yu et al., A biomimetic hierarchical nanointerface orchestrates macrophage polarization and mesenchymal stem cell recruitment to promote endogenous bone regeneration. ACS Nano **13**(6), 6581–6595 (2019). 10.1021/acsnano.9b00489 31125522

[47] S. Reinke, S. Geissler, W.R. Taylor, K. Schmidt-Bleek, K. Juelke et al., Terminally differentiated CD8(+) T cells negatively affect bone regeneration in humans. Sci Transl Med. **5**(177), 177–136 (2013). 10.1126/scitranslmed.3004754 23515078

[48] Y. Liu, L. Wang, T. Kikuiri, K. Akiyama, C. Chen et al., Mesenchymal stem cell-based tissue regeneration is governed by recipient t lymphocytes via IFN-gamma and TNF-alpha. Nat. Med. **17**(12), 1594–1601 (2011). 10.1038/nm.2542 PMC3233650 22101767

[49] D. Toben, I. Schroeder, T. El Khassawna, M. Mehta, J.E. Hoffmann et al., Fracture healing is accelerated in the absence of the adaptive immune system. J. Bone Miner. Res. **26**(1), 113–124 (2011). 10.1002/jbmr.185 20641004

[50] L. Chung, D.R. Maestas Jr., F. Housseau, J.H. Elisseeff, Key players in the immune response to biomaterial scaffolds for regenerative medicine. Adv. Drug Deliv. Rev. **114**, 184–192 (2017). 10.1016/j.addr.2017.07.006 28712923

[51] K.A. Daly, S. Liu, V. Agrawal, B.N. Brown, S.A. Johnson et al., Damage associated molecular patterns within xenogeneic biologic scaffolds and their effects on host remodeling. Biomaterials **33**(1), 91–101 (2012). 10.1016/j.biomaterials.2011.09.040 21967802

[52] B. Shokouhi, C. Coban, V. Hasirci, E. Aydin, A. Dhanasingh et al., The role of multiple toll-like receptor signalling cascades on interactions between biomedical polymers and dendritic cells. Biomaterials **31**(22), 5759–5771 (2010). 10.1016/j.biomaterials.2010.04.015 20452017

[53] K. Lynch, O. Treacy, J.Q. Gerlach, H. Annuk, P. Lohan et al., Regulating immunogenicity and tolerogenicity of bone marrow-derived dendritic cells through modulation of cell surface glycosylation by dexamethasone treatment. Front. Immunol. **8**, 1427 (2017). 10.3389/fimmu.2017.01427 PMC5670353 29163502

[54] S.E. Skrabalak, J. Chen, Y. Sun, X. Lu, L. Au et al., Gold nanocages: Synthesis, properties and applications. Acc. Chem. Res. **41**(12), 1587–1595 (2008). 10.1021/ar800018v PMC2645935 18570442

[55] X. Zhu, W. Feng, J. Chang, Y.W. Tan, J. Li et al., Temperature-feedback upconversion nanocomposite for accurate photothermal therapy at facile temperature. Nat. Commun. **7**, 10437 (2016). 10.1038/ncomms10437 PMC4742858 26842674

[56] L.K. Kozicky, L.M. Sly, Depletion and reconstitution of macrophages in mice. Methods Mol. Biol. **1960**, 101–112 (2019). 10.1007/978-1-4939-9167-9_9 30798525

[57] L. Tang, T.A. Jennings, J.W. Eaton, Mast cells mediate acute inflammatory responses to implanted biomaterials. Proc. Natl. Acad. Sci. USA **95**(15), 8841–8846 (1998). 10.1073/pnas.95.15.8841 PMC21164 9671766

[58] M.N. Michalski, L.E. Zweifler, B.P. Sinder, A.J. Koh, J. Yamashita et al., Clodronate-loaded liposome treatment has site-specific skeletal effects. J. Dent. Res. **98**(4), 459–467 (2019). 10.1177/0022034518821685 PMC6429666 30626255

[59] A. Hotblack, S. Seshadri, L. Zhang, S. Hamrang-Yousefi, R. Chakraverty et al., Dendritic cells cross-present immunogenic lentivector-encoded antigen from transduced cells to prime functional T cell immunity. Mol. Ther. **25**(2), 504–511 (2017). 10.1016/j.ymthe.2016.11.001 PMC5368353 28153097

[60] J. Li, J. Tan, M.M. Martino, K.O. Lui, Regulatory t-cells: Potential regulator of tissue repair and regeneration. Front. Immunol. **9**, 585 (2018). 10.3389/fimmu.2018.00585 PMC5890151 29662491

[61] S. Chen, L. Fang, W. Guo, Y. Zhou, G. Yu, W. Li et al., Control of treg cell homeostasis and immune equilibrium by lkb1 in dendritic cells. Nat. Commun. **9**(1), 5298 (2018). 10.1038/s41467-018-07545-8 PMC6294005 30546010

[62] G.S. Selders, A.E. Fetz, M.Z. Radic, G.L. Bowlin, An overview of the role of neutrophils in innate immunity, inflammation and host-biomaterial integration. Regen Biomater. **4**(1), 55–68 (2017). 10.1093/rb/rbw041 PMC5274707 28149530

