# Correction: Dual-Wavelength Photosensitive Nano-in-Micro Scaffold Regulates Innate and Adaptive Immune Responses for Osteogenesis

**DOI:** 10.1007/s40820-026-02338-x

**Published:** 2026-09-30

**Authors:** Qin Zhao, Miusi Shi, Chengcheng Yin, Zifan Zhao, Jinglun Zhang, Jinyang Wang, Kailun Shen, Lingling Zhang, Hua Tang, Yin Xiao, Yufeng Zhang

**Affiliations:** 1 The State Key Laboratory Breeding Base of Basic Science of Stomatology (Hubei-MOST) and Key Laboratory of Oral Biomedicine, School and Hospital of Stomatology, Wuhan University, Ministry of Education, Wuhan, 430079 People’s Republic of China; 2 Medical Research Institute, School of Medicine, Wuhan University, Wuhan, 430071 People’s Republic of China; 3 Institute of Immunology, Shandong First Medical University & Shandong Academy of Medical Sciences, Taian, 271000 People’s Republic of China; 4 Institute of Health and Biomedical Innovation & Australia-China Centre for Tissue Engineering and Regenerative Medicine, Queensland University of Technology, Kelvin Grove, QLD 4059 Australia

**Correction to: Nano-Micro Letters (2021) 13:28** **https://doi.org/10.1007/s40820-020-00540-z**

Following publication of the original article [1], the authors identified several inadvertent errors in Figs. 1 and 3.

In Fig. 1c, due to an oversight during the final assembly of the figures, the authors mistakenly used a photograph from another group for the BCP-8W-CD146 panel. The original and correct photograph has now been provided. This error was purely layout-related; it does not affect the quantitative data, statistical analyses, or the scientific conclusions of the study.

In Fig. 3, three minor graphical errors occurred during the final typesetting and vector-formatting phase, which were unfortunately missed by the authors during proofreading. In Fig. 3i, the y-axis label for the *β*-TCP + DC2.4 group should read "1.5 K" instead of "1.5". In Fig. 3j, a minor column misalignment occurred in the BCP + 2.4 group. In Fig. 3l, the maximum value of the y-axis should be "20" instead of "25".

The correct Figs. 1 and 3 have been provided in this Correction.

The incorrect Fig. 1 is:

**Fig. 1:**
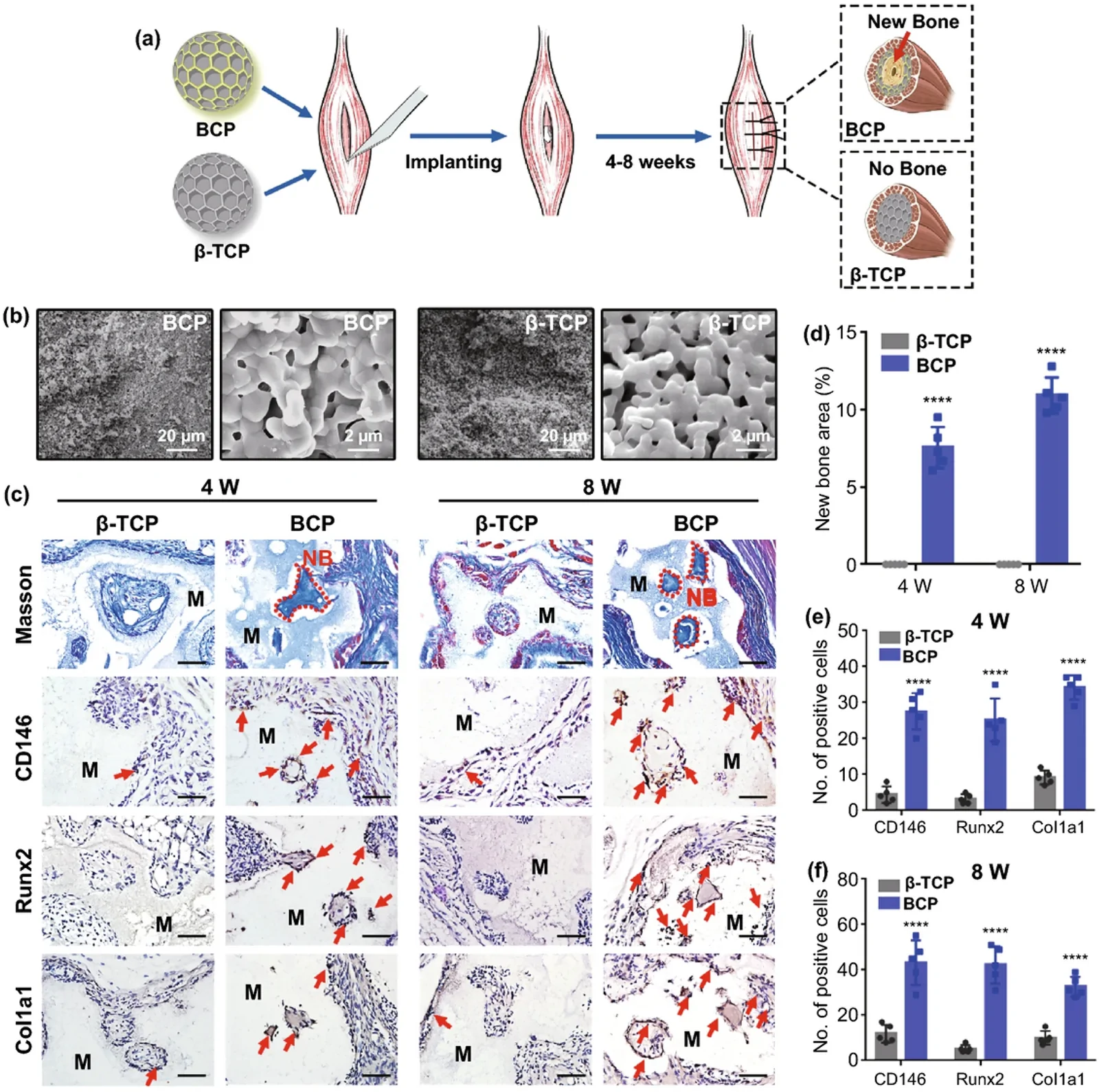
**a** Schematic diagram of BCP and *β*-TCP implantation and new bone formation. **b** Scheme, morphology and nanostructure of BCP and *β*-TCP observed by SEM. **c** Masson staining and IHC staining (CD146, Runx2 and Col1a1: MSC and osteoblast markers, red arrow) of implant area of BCP and *β*-TCP in vivo after 4 weeks (4 W) and 8 weeks (8 W) (the red dash line shows the new bone formation area; NB, new bone; M, material. Scale bar = 100 μm). **d**–**f** Semiquantification of new bone area and positively stained cells in (**d**). n = 5, ****P < 0.0001

The correct Fig. 1 is:

**Fig. 1:**
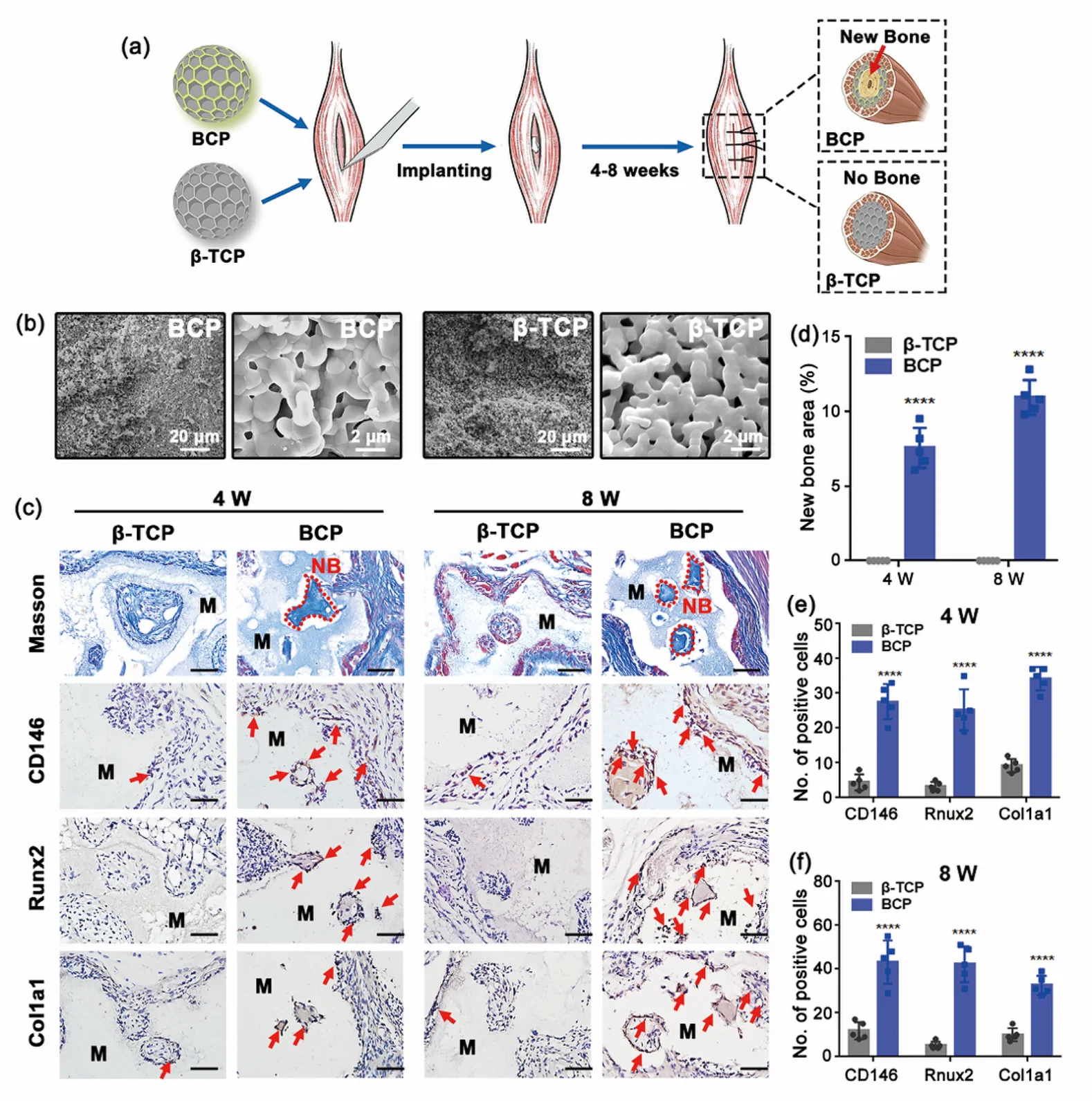
**a** Schematic diagram of BCP and *β*-TCP implantation and new bone formation. **b** Scheme, morphology and nanostructure of BCP and *β*-TCP observed by SEM. **c** Masson staining and IHC staining (CD146, Runx2 and Col1a1: MSC and osteoblast markers, red arrow) of implant area of BCP and *β*-TCP in vivo after 4 weeks (4 W) and 8 weeks (8 W) (the red dash line shows the new bone formation area; NB, new bone; M, material. Scale bar = 100 μm). **d**–**f** Semiquantification of new bone area and positively stained cells in (**d**). n = 5, ****P < 0.0001

The incorrect Fig. 3 is:

**Fig. 3:**
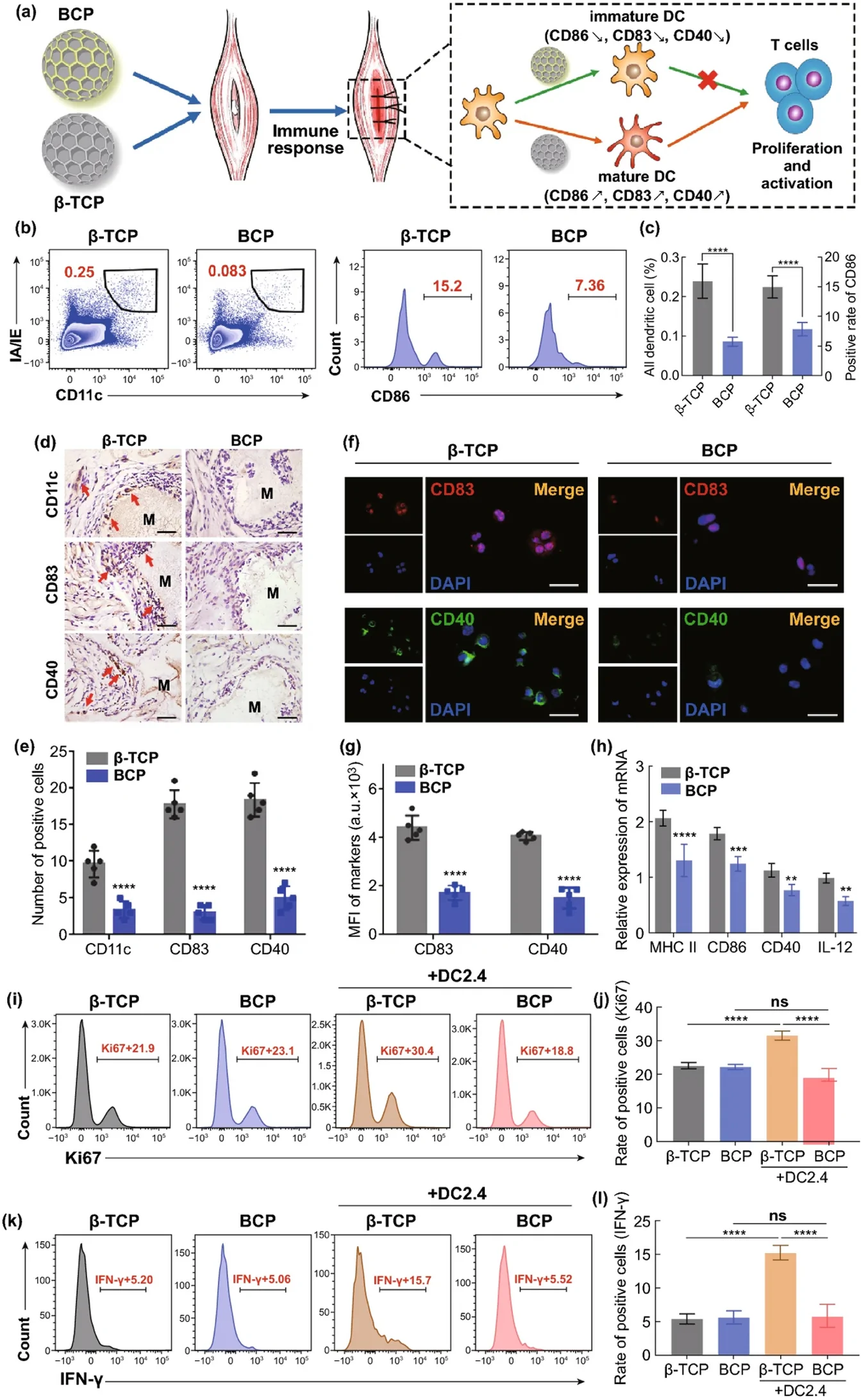
**a** Schematic diagram of biomaterial-mediated DC maturation and T cell activation. **b** FACS of all DCs (CD11c + , IA/IE +) and mature DCs (CD11c + , IA/IE + , CD86 +) recruitment at 2 weeks after biomaterials implantation in vivo. **c** Semiquantification of gating strategy cells in (B). n = 6, ****P < 0.0001. **d** IHC staining of CD11c—DC marker and CD83/CD40—mature DC markers at 4 weeks after biomaterials implantation in vivo. Red arrow, positive cell. Scale bar = 100 μm. M, material. **e** Semiquantification of positively stained cells in (**d**). n = 5, ****P < 0.0001. **f** Immunofluorescent (IF) staining of CD83 (red), CD40 (green) and nuclei (blue) after DCs seeded on the BCP or *β*-TCP for 24 h. Scale bar = 5 μm. **g** Semiquantification of positively stained cells in (**f**). n = 5, ****P < 0.0001, ns: not significant. **h** Relative mRNA expressions of DC maturation genes (MHC II, CD86, CD40, IL-12). n = 3, **P < 0.01, ***P < 0.001, ****P < 0.0001. **i-l** FACS of T cell proliferation (Ki67 +) and activation (IFN-γ) and semiquantification of positive cell rate. n = 6, ***P < 0.001, ****P < 0.0001, ns: not significant

The correct Fig. 3 is:

**Fig. 3:**
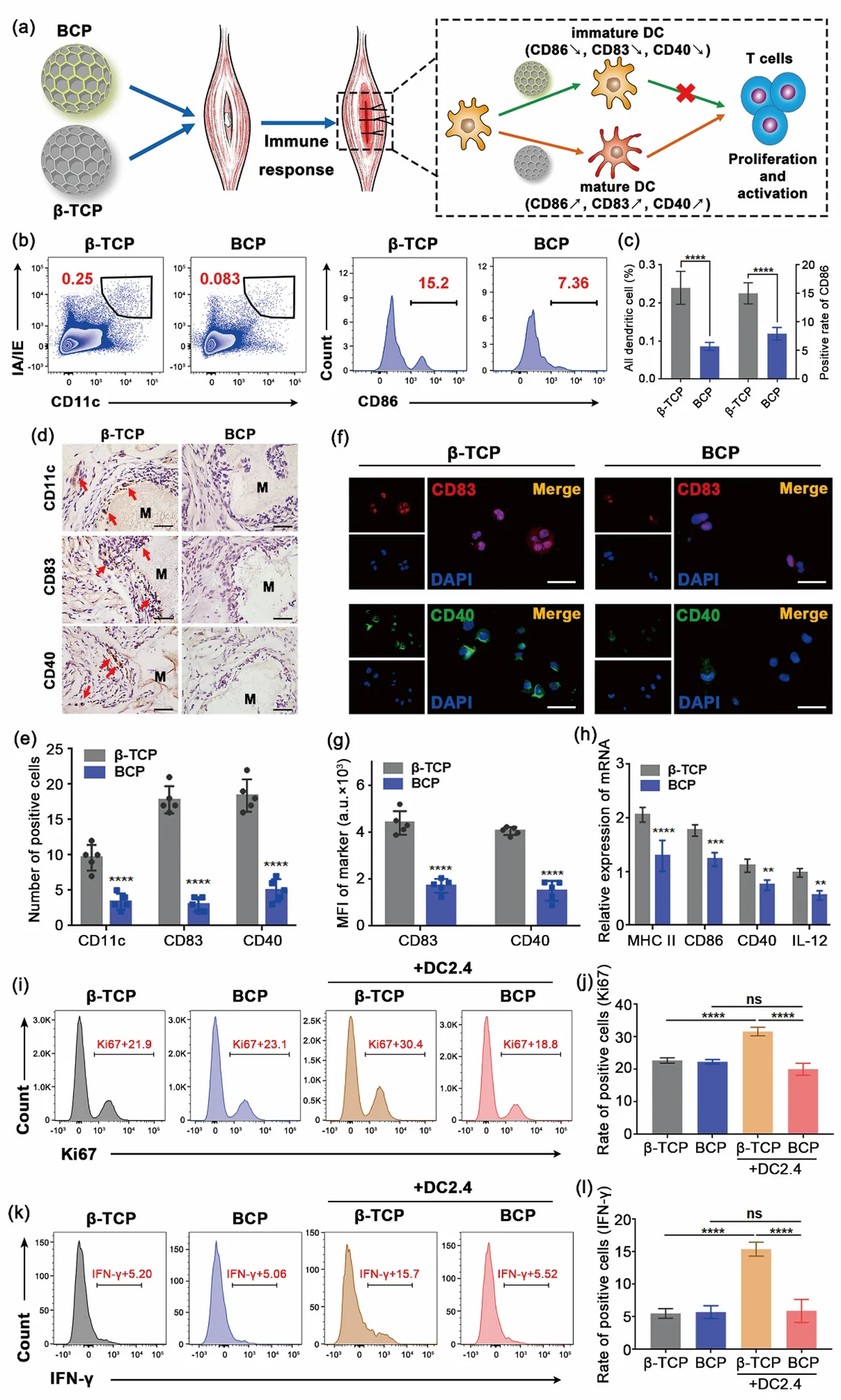
**a** Schematic diagram of biomaterial-mediated DC maturation and T cell activation. **b** FACS of all DCs (CD11c + , IA/IE +) and mature DCs (CD11c + , IA/IE + , CD86 +) recruitment at 2 weeks after biomaterials implantation in vivo. **c** Semiquantification of gating strategy cells in (B). n = 6, ****P < 0.0001. **d** IHC staining of CD11c—DC marker and CD83/CD40—mature DC markers at 4 weeks after biomaterials implantation in vivo. Red arrow, positive cell. Scale bar = 100 μm. M, material. **e** Semiquantification of positively stained cells in (**d**). n = 5, ****P < 0.0001. **f** Immunofluorescent (IF) staining of CD83 (red), CD40 (green) and nuclei (blue) after DCs seeded on the BCP or *β*-TCP for 24 h. Scale bar = 5 μm. **g** Semiquantification of positively stained cells in (**f**). n = 5, ****P < 0.0001, ns: not significant. **h** Relative mRNA expressions of DC maturation genes (MHC II, CD86, CD40, IL-12). n = 3, **P < 0.01, ***P < 0.001, ****P < 0.0001. **i-l** FACS of T cell proliferation (Ki67 +) and activation (IFN-γ) and semiquantification of positive cell rate. n = 6, ***P < 0.001, ****P < 0.0001, ns: not significant

The original article [1] has been corrected.
