# Supplementary material for: Dual-Wavelength Photosensitive Nano-in-Micro Scaffold Regulates Innate and Adaptive Immune Responses for Osteogenesis
Source: Nanomicro Lett. 2020 Nov 21;13:28. doi: 10.1007/s40820-020-00540-z (PMC8187671; doi:10.1007/s40820-020-00540-z)
Supplement: Supplementary file 1 — Supplementary file1 (PDF 1654 kb) [file 40820_2020_540_MOESM1_ESM.pdf]

Supporting Information for

Dual-Wavelength Photosensitive Nano-in-Micro Scaffold Regulates Innate and Adaptive Immune Responses for Osteogenesis

Qin Zhao[1], Miusi Shi[1], Chengcheng Yin[1], Zifan Zhao[1], Jinglun Zhang[1], Jinyang Wang[1], Kailun Shen[1], Lingling Zhang[1, 2], Hua Tang[3], Yin Xiao[4], Yufeng Zhang[1, 2, *]

[1]The State Key Laboratory Breeding Base of Basic Science of Stomatology (Hubei-MOST) and Key Laboratory of Oral Biomedicine, Ministry of Education, School and Hospital of Stomatology, Wuhan University, Wuhan 430079, People's Republic of China

[2]Medical Research Institute, School of Medicine, Wuhan University, Wuhan 430071, People's Republic of China

[3]Institute of immunology, Shandong First Medical University & Shandong Academy of Medical Sciences, Taian 271000, People's Republic of China

[4]Institute of Health and Biomedical Innovation & Australia-China Centre for Tissue Engineering and Regenerative Medicine, Queensland University of Technology, Kelvin Grove, 4059 QLD, Australia

*Corresponding author. E-mail: zyf@whu.edu.cn (Yufeng Zhang)

Supplementary Figures and Table

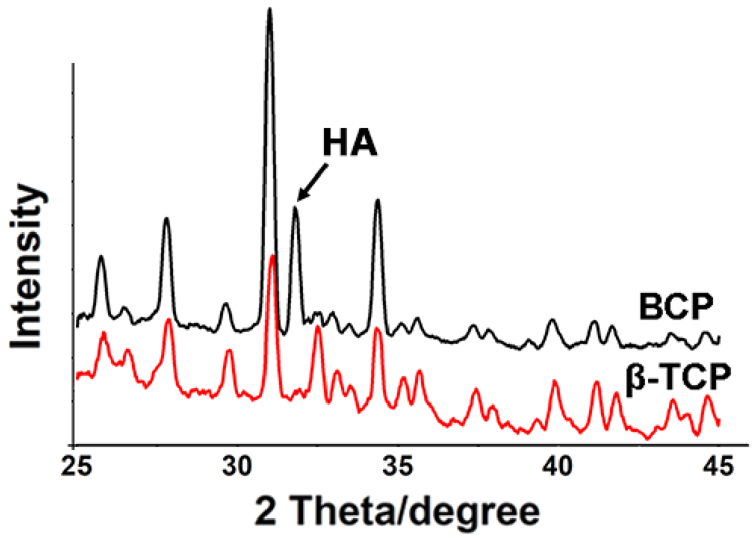

Fig. S1 XRD analysis showing the composition of BCP and β-TCP ceramics with their characteristic peaks indicated. The black line is the absorption peak curve of BCP, and the red is β-TCP. In addition to the peaks similar to β-TCP, there is a characteristic peak of HA phase in the BCP absorption peak curve

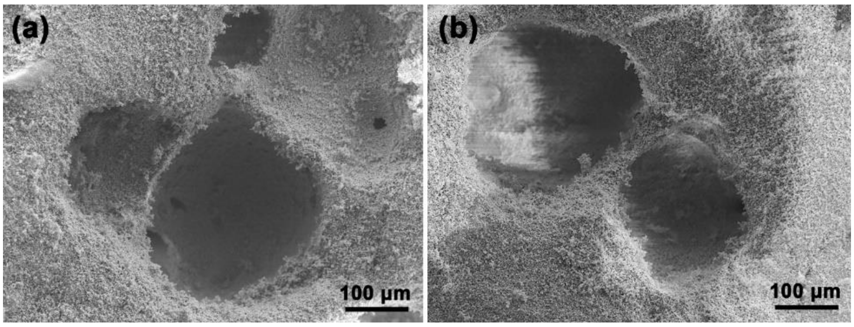

Fig. S2 SEM images of BCP and β-TCP in low magnification. **a** BCP scaffold. **b** β-TCP scaffold

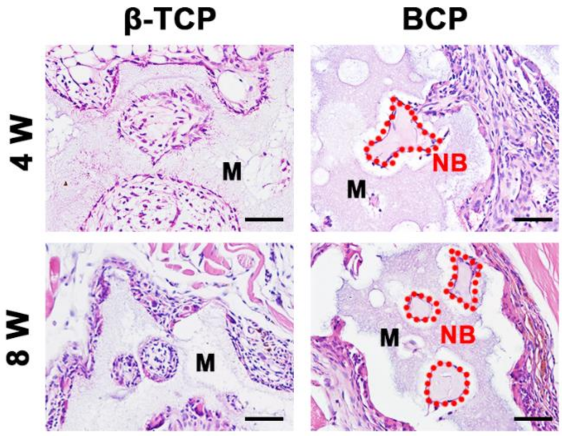

Fig. S3 H&E staining of implant area of BCP and β-TCP in vivo after 4 weeks (4 W) and 8 weeks (8 W). BCP can induce new bone formation in vivo. (the red dash line shows the new bone formation area; NB, new bone; M, material; Scale bar = 100 μm)

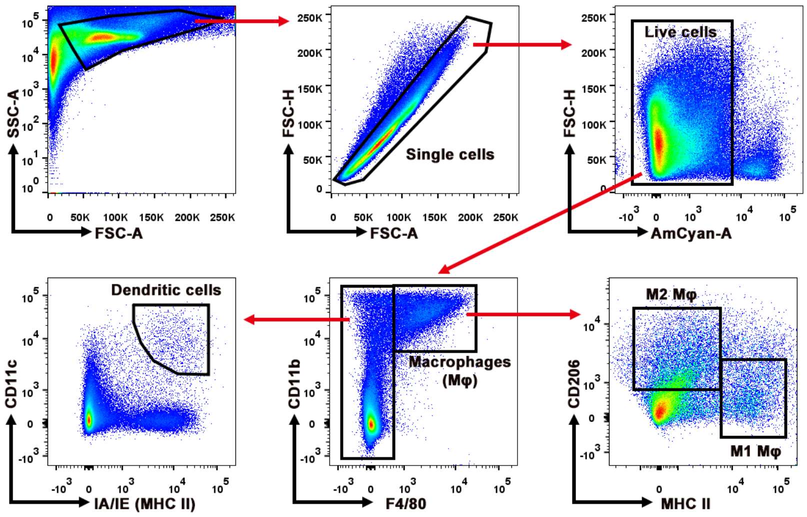

Fig. S4 Gating strategy of FCM. First, use FSC-A and FSC-H to select effective single cells, and then remove the dead cells (AmCyan-A+). Among live cells, F4/80 and CD11b double-positive cells are macrophages, which are further divided into M1 macrophages (MHC II+) and M2 macrophages (CD206+). Among the cells from which macrophages are removed, CD11c and IA/IE double-positive cells are DCs

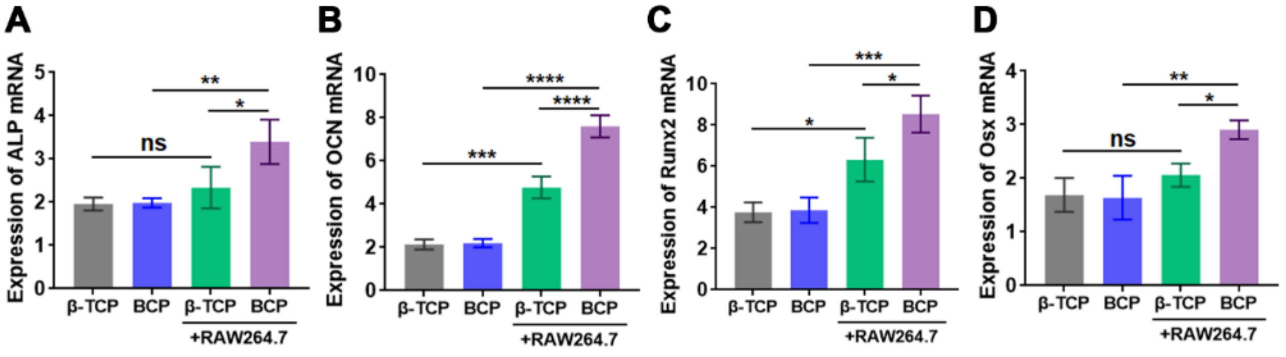

Fig. S5 Relative mRNA expressions of osteogenic genes ALP (A), OCN (B), Runx2 (C), Osx (D). BCP-stimulated macrophages are beneficial to the osteogenic gene expression of MSCs. n=3, $*P < 0.05$, $**P < 0.01$, $***P < 0.001$, $****P < 0.0001$. ns: not significant

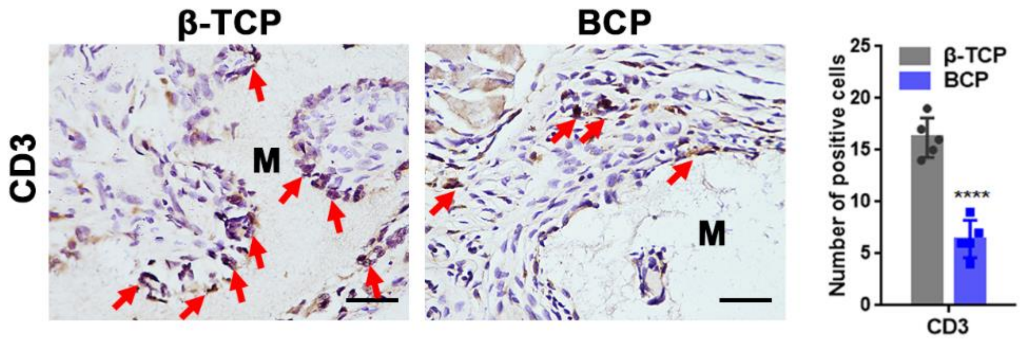

Fig. S6 Immuohistochemical (IHC) staining (CD3: T cell marker, red arrow) of implant area of BCP and β-TCP in vivo after 4 weeks (M, material), and the semiquantification of positively stained cells (CD3). There are fewer T cells around the BCP after the implantation of biomaterials. Scale bar = 100 µm. n=5, $****P < 0.0001$

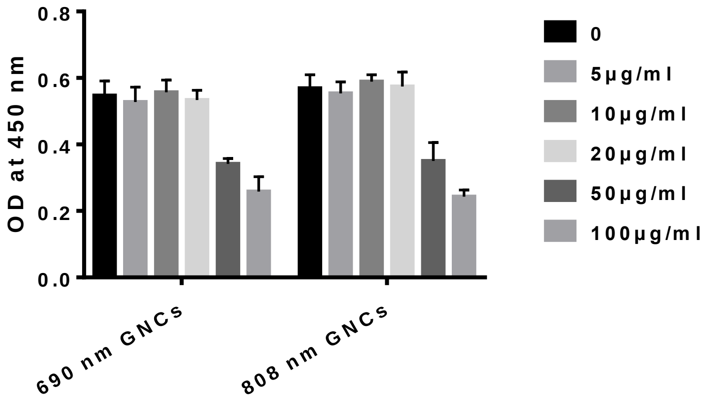

Fig. S7 Cell viability at different concentrations of 690 nm GNCs and 808 nm GNCs. The 0-20 µg mL^{-1} GNCs have little effect on cell viability, and cell viability decreases when the concentration of GNCs is 50 µg mL^{-1} or above. RAW264.7 cell viability evaluated by CCK8 assay after incubation with different concentrations of them for 72 h

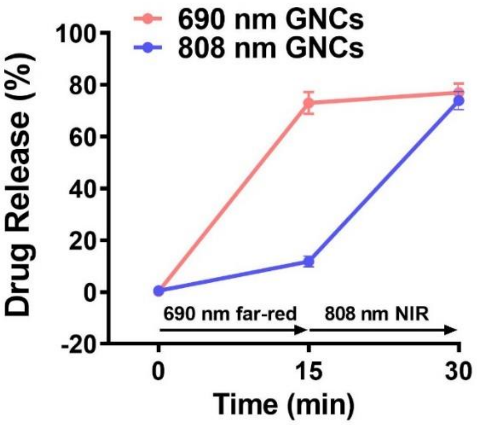

Fig. S8 Fluorescein methylene blue (MB) was used as a model drug to load into 690 nm GNCs, and Rhodamine B as another model drug to load into 808 nm GNCs. 690 nm far red or 808 nm NIR controlled release of fluorescein MB load in 690 nm GNCs and 808 nm GNCs. The two kinds of GNCs were irradiated together with 690 nm far-red for 15 min first, and then with 808 nm NIR for 15 min later. 690 nm far-red or 808 nm NIR controlled dual release of fluorescein MB and Rhodamine B. The power density of irradiation was 1.0 W cm^{-2}.

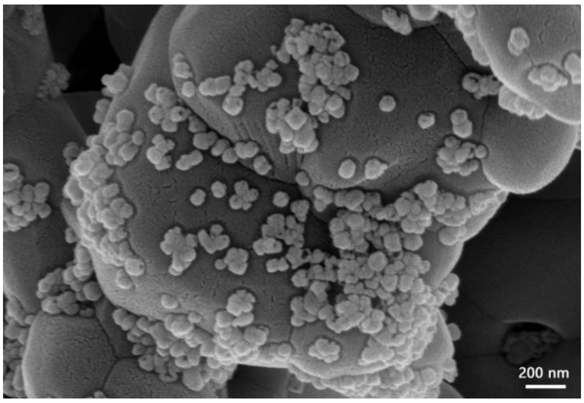

Fig. S9 SEM images of BCP-GNCs after 7 days of immersion in simulate body fluid (SBF)

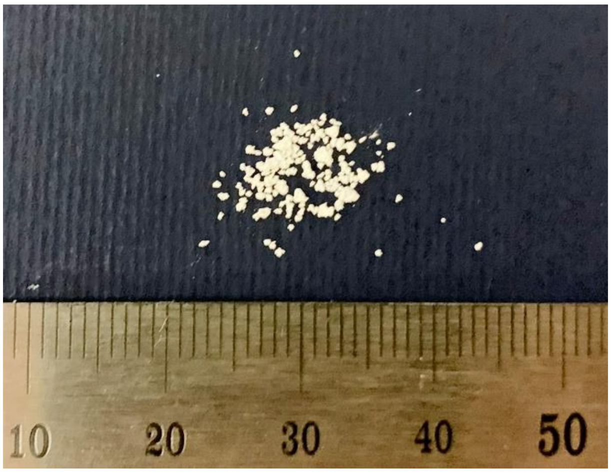

Fig. S10 Visual photo of BCP. They were particles, and each grid of the ruler was 1 mm

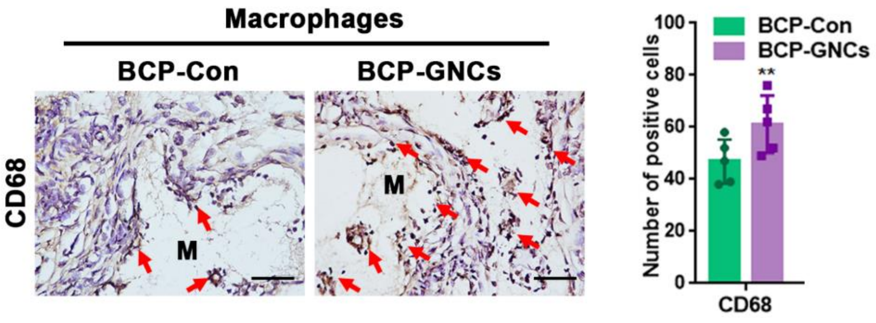

Fig. S11 IHC staining of total macrophages (CD68) under the BCP-GNCs implant *in vivo*, and the semiquantification of positively stained cells (CD68). There are more macrophages (CD68) around the BCP-GNCs after the irradiations. Scale bar = 100 μm. Red arrow, positive cells. M, material. n=5, ***P* < 0.01

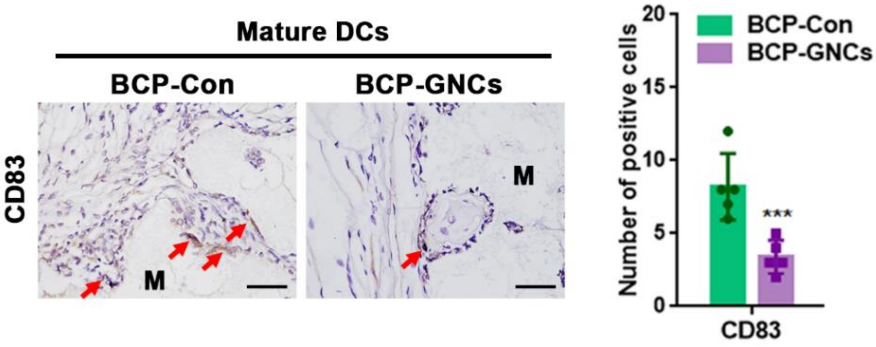

Fig. S12 IHC staining of total mature DCs (CD83) under the BCP-GNCs implant in vivo, and the semiquantification of positively stained cells (CD83). There are fewer mature DCs (CD83) around the BCP-GNCs after the irradiations. Scale bar = 100 μm. Red arrow, positive cells. M, material. n=5, ***P < 0.001

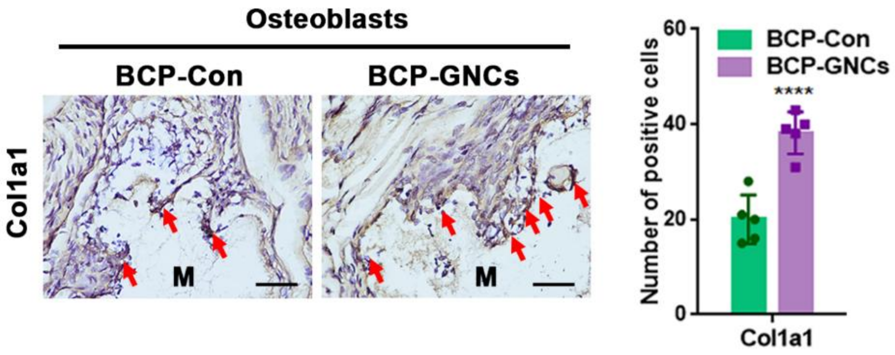

Fig. S13 IHC staining of total osteoblasts (Col1a1) under the BCP-GNCs implant *in vivo*, and the semiquantification of positively stained cells (Col1a1). There are more osteoblasts (Col1a1) around the BCP-GNCs after the irradiations. Scale bar = 100 μm. Red arrow, positive cells. M, material. n=5, *****P* < 0.0001

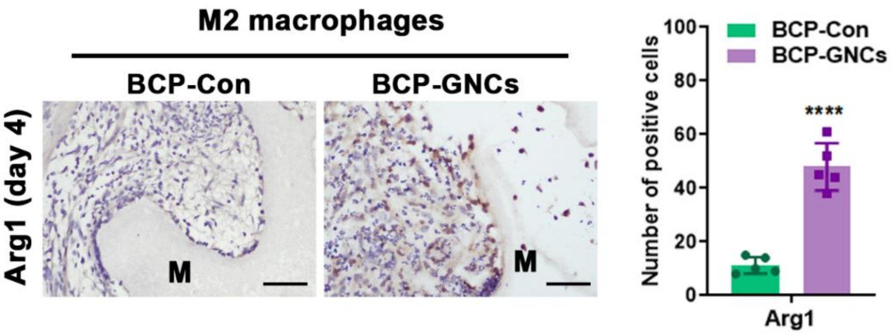

Fig. S14 IHC staining of M2 macrophages (Arg1) after the release of IL-4 (at day 4), and the semiquantification of positively stained cells (Arg1). There are more M2 macrophages (Arg1) around the BCP-GNCs after IL-4 release. Scale bar = 100 μm. M, material. n=5, ****$P < 0.0001$

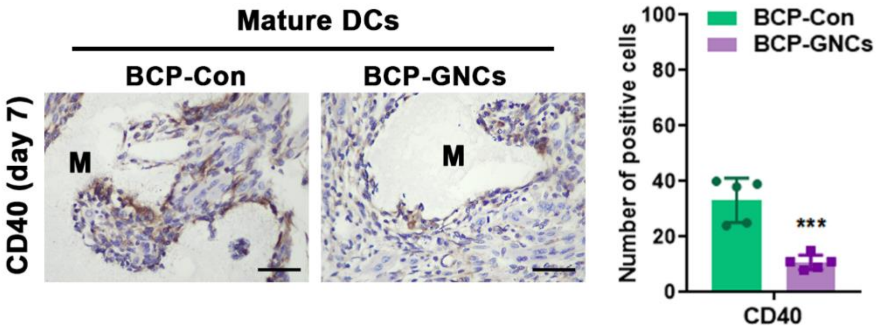

Fig. S15 IHC staining of mature DCs (CD40) after the release of DXMS (at day 7), and the semiquantification of positively stained cells (CD40). There are fewer mature DCs (CD40) around the BCP-GNCs after DXMS release. Scale bar = 100 μm. M, material. n=5, ***$P < 0.001$

Table S1 Primer sequences of target genes

Gene	Forward primer sequence	Reverse primer sequence
ALP	5’-TGGACGGTGAACGGGAAAAT-3’	5’-TAGTTCTGCTCATGGACGCC-3’
Runx2	5’-CCCAGTATGAGAGTAGGTGTCC-3’	5’-GGGTAAGACTGGTCATAGGACC-3’
OCN	5’-CGCTACCTGTATCAATGGCTGG-3’	5’-CTCCTGAAAGCCGATGTGGTCA-3’
Osx	5’-CAACCTGCTAGAGATCTGAG-3’	5’-TGCAATAGGAGAGAGCGA-3’
IL-10	5’-GGTTGCCAAGCCTTATCGGA-3’	5’-ACCTGCTCCACTGCCTTGCT-3’
IL-12	5’-GGAAGCACGGCAGCAGAATA-3’	5’-AACTTGAGGGAGAAGTAGGAATGG-3’
TNF-α	5’-TGTCTCAGCCTCTTCTCATT-3’	5’-TGATCTGAGTGTGAGGGTCT-3’
CD86	5’-CAGAACTTACGGAAGCACCCA-3’	5’-ATAAGCTTGCGTCTCCACGG-3’
CD40	5’-CCTGCCCAGTCGGCTTCT-3’	5’-GTCCAAGGGTGACATTTTTCG-3’
MHC II	5’-TGGTGACACTGGGACATTCATC-3’	5’-CAGTCTGTCCCCCTGCTAAG-3’
iNOS	5’-AAGATGGCCTGGAGGAATGC-3’	5’-TGCTGTGCTACAGTTCCGAG-3’
Arg1	5’-CAGCACTGAGGAAAGCTGGT-3’	5’-CAGACCGTGGGTTCTTCACA-3’
GAPDH	5’-GCACCGTCAAGGCTGAGAAC-3’	5’-TGGTGAAGACGCCAGTGGA-3’
